# Critical Role of MetR/MetB/MetC/MetX in Cysteine and Methionine Metabolism, Fungal Development, and Virulence of Alternaria alternata

**DOI:** 10.1128/AEM.01911-20

**Published:** 2021-01-29

**Authors:** Yunpeng Gai, Lei Li, Haijie Ma, Brendan K. Riely, Bing Liu, Hongye Li

**Affiliations:** a Key Lab of Molecular Biology of Crop Pathogens and Insects, Ministry of Agriculture, Institute of Biotechnology, Zhejiang University, Hangzhou, China; b Department of Plant Pathology, University of California, Davis, Davis, California, USA; c School of Agriculture and Food Sciences, Zhejiang Agriculture & Forestry University, Hangzhou, China; d College of Forestry, Nanjing Forestry University, Nanjing, China; University of Illinois at Urbana-Champaign

**Keywords:** *Alternaria alternata*, cysteine and methionine metabolism, gene function, oxidative stress tolerance, pathogenicity, transcriptome analysis

## Abstract

Methionine is a unique sulfur-containing amino acid which plays an important role in biological protein synthesis and various cellular processes. Here, we characterized the biological functions of *AaMetB*, *AaMetC*, and *AaMetX* in the tangerine pathotype of Alternaria alternata. Morphological analysis showed that the mutants lacking *AaMetB*, *AaMetC*, or *AaMetX* resulted in less aerial hyphae and fewer conidia in artificial media. Pathogenicity analysis showed that *AaMetB*, *AaMetC*, and *AaMetX* are required for full virulence. The defects in vegetative growth, conidiation, and virulence of *MetB*, *MetC*, and *MetX* deletion mutants (hereafter called Δ*MetB*, Δ*MetC*, and Δ*MetX*) can be restored by exogenous methionine and homocysteine, indicating that *AaMetB*, *AaMetC*, and *AaMetX* are required for methionine biosynthesis. However, exogenous cysteine restored the growth and virulence defects of Δ*MetR* but not Δ*MetB*, Δ*MetC*, and Δ*MetX*, suggesting that *AaMetR* is essential for cysteine biosynthesis. Oxidant sensitivity assay showed that only *ΔMetR* is sensitive to H_2_O_2_ and many reactive oxygen species (ROS)-generating compounds, indicating that *AaMetR* is essential for oxidative tolerance. Interestingly, fungicide indoor bioassays showed that only the Δ*MetR* mutant is susceptive to chlorothalonil, a fungicide that could bind to the cysteine of glyceraldehyde-3-phosphate dehydrogenase. Comparative transcriptome analysis showed that the inactivation of *MetB*, *MetC*, *MetX*, and *MetR* significantly affected the expression of methionine metabolism-related genes. Moreover, the inactivation of *AaMetR* significantly affected the expression of many genes related to glutathione metabolism, which is essential for ROS tolerance. Taken together, our study provides genetic evidence to define the critical roles of *AaMetB*, *AaMetC*, *AaMetX*, and *AaMetR* in cysteine and methionine metabolism, fungal development, and virulence of Alternaria alternata.

**IMPORTANCE** The transcription factor MetR, regulating methionine metabolism, is essential for ROS tolerance and virulence in many phytopathogenic fungi. However, the underlying regulatory mechanism of MetR involved in this process is still unclear. In the present study, we generated *AaMetB*, *AaMetC*, and *AaMetX* deletion mutants and compared these mutants with *AaMetR*-disrupted mutants. Interestingly, we found that *AaMetB*, *AaMetC*, and *AaMetX* are required for vegetative growth, conidiation, and pathogenicity in Alternaria alternata but not for ROS tolerance and cysteine metabolism. Furthermore, we found that MetR is involved in the biosynthesis of cysteine, which is an essential substrate for the biosynthesis of methionine and glutathione. This study emphasizes the critical roles of *MetR*, *MetB*, *MetC*, and *MetX* in the regulation of cysteine and methionine metabolism, as well as the cross-link with glutathione-mediated ROS tolerance in phytopathogenic fungi, which provides a foundation for future investigations.

## Abstract (precis)

The transcription factor METR, regulating methionine metabolism, is essential for ROS tolerance and virulence in many phytopathogenic fungi. However, the underlying regulatory mechanism of METR involved in this process is still unclear.

## INTRODUCTION

Alternaria alternata (Fr.) Keissler is an important necrotroph with a broad host range. A. alternata is comprised of at least seven pathotypes, each of which can secrete a unique host-selective toxin (HST), kill host cells before the invasion, and extensively absorb nutrients from dead tissues (1, 2). In citrus plants, the tangerine pathotype and rough lemon pathotype are two completely different pathotypes of A. alternata that have been identified to date. A. alternata tangerine pathotype can produce ACT (Alternaria citri tangerine) toxin, which can cause severe brown spots on tangerines (Citrus reticulata Blanco), grapefruit (Citrus paradisi Merced.), sweet orange (Citrus sinensis [L.] Osbeck), and their hybrids. In contrast, the rough lemon pathotype is known to produce ACRL (Alternaria citri rough lemon) toxin, which is toxic to rough lemon but not tangerine (2–4). The pathogenicity of A. alternata tangerine pathotype mainly relies on the host-selective ACT toxin, which is regulated by multiple copies of *ACTT* gene clusters located on less than 2.0 Mb of conditionally dispensable chromosome (CDC) (5, 6). Intriguingly, genetic inactivation of any *ACTT* gene will block the biosynthesis of ACT toxin and lead to complete loss of pathogenicity but will not affect fungal growth and conidiation (5–7). In addition to HST biosynthesis, the ability to detoxify host-generating reactive oxygen species (ROS) regulated by *AaYap1*, *AaHog1*, and *AaSsn7* is essential for the full virulence of A. alternata (8–11). ROS are reactive molecules mainly generated by NADPH oxidase complex (including subunits NOXA, NOXB, and NOXR) (12, 13). Fungal mutants lacking either *AaNoxA*, *AaNoxB*, *AaYap1*, *AaHog1*, or *AaSkn7* displayed increased sensitivity to oxidants and reduced virulence to citrus (8–11). Recent studies have shown that *AaYap1* and *AaHog1* transcriptionally regulate *AaGPx3*, *AaGlr1*, *AaTsa1*, and *AaTrr1*, which are the major antioxidant systems in A. alternata. AaTSA1 can jointly work with AaTRR1 to interconvert reduced thioredoxin (TRX^[red]^) and oxidized thioredoxin (TRX^[ox]^) in the thioredoxin system, which is a crucial process of ROS detoxification. Fungal mutants lacking *AaTsa1* or *AaTrr1* in A. alternata were more susceptible to oxidative stress and less pathogenic to citrus (14, 15). Therefore, the NADPH oxidase complex (NOXA, NOXB, and NOXR), the YAP1 antioxidant system (YAP1, HOG1, and SKN7), and its downstream glutathione/thioredoxin system (glutathione peroxidase oxidase [GPx3], glutathione-disulfide reductase [GLR1], thioredoxin peroxidase [TSA1], and thioredoxin reductase [TRR1]) constitute a complex antioxidant network that regulates ROS detoxification (15).

**FIG 1:**
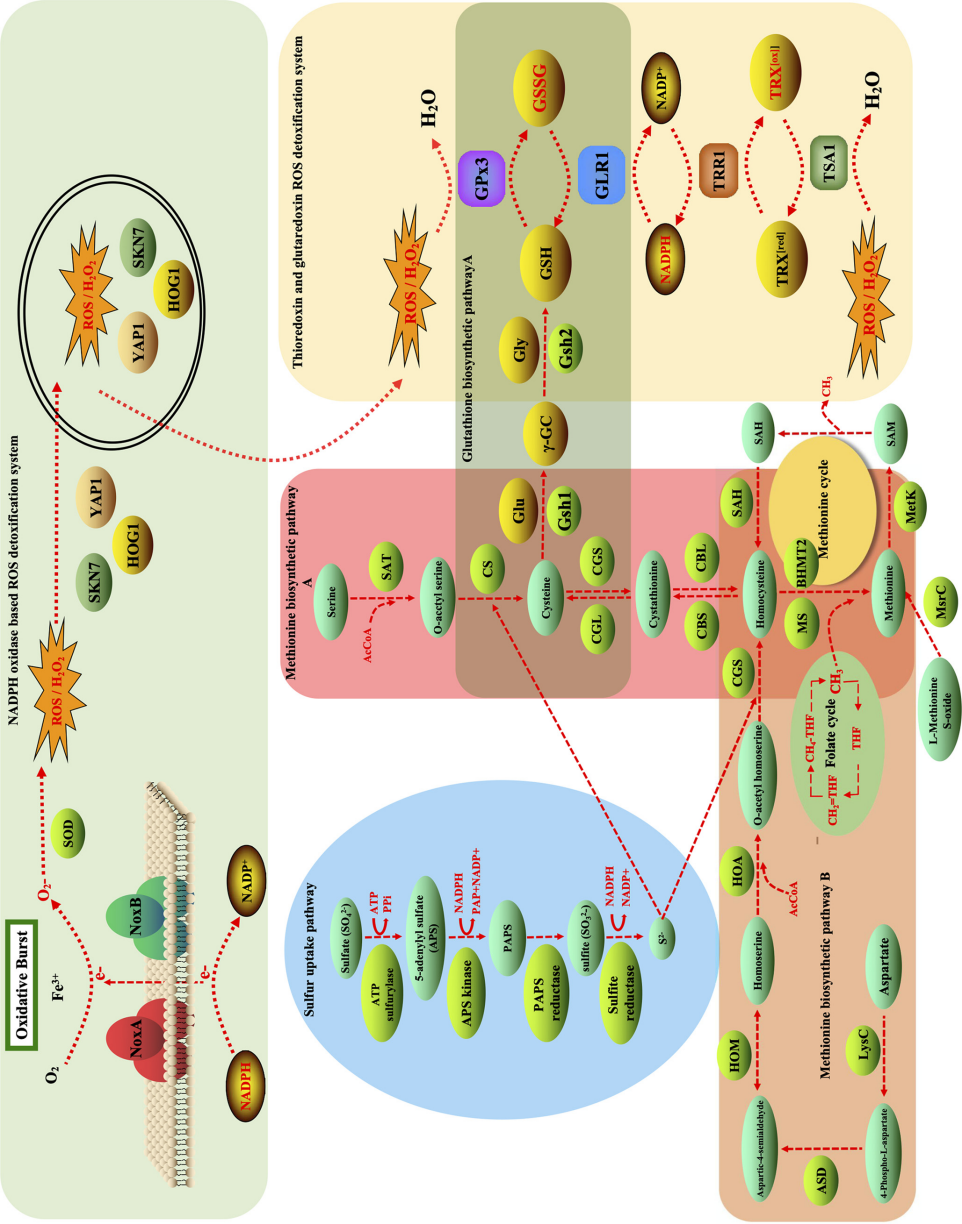
Proposed regulatory network of sulfur uptake, methionine biosynthesis pathway, and fungal antioxidant system that involves NADPH oxidase (NOX), YAP1, HOG1, SKN7, GPx3, GLR1, TRR1, and TSA1 in Alternaria alternata. CGS represents cystathionine gamma-synthase encoded by the *MetB* gene (GenBank accession no. MN812273). CBL represents cysteine S-conjugate beta-lyase (KEGG accession no. K01760) encoded by the *MetC* gene (MN812274). HOA represents homoserine O-acetyltransferase (KEGG accession no. K00641) encoded by the *MetX* gene (MN812275). The superoxide (O_2_^−^) produced by NADPH oxidase can be converted to H_2_O_2_ in a reaction catalyzed by superoxide dismutase (SOD). GPx3 and glutathione reductase (GLR1) can detoxify H_2_O_2_ in the glutathione cycling between reduced glutathione (GSH) and oxidized glutathione (GSSG). TSA1 and thioredoxin reductase (TRR1) can detoxify H_2_O_2_ in the thioredoxin cycling between reduced thioredoxin (TRX^[red]^) and oxidized thioredoxin (TRX^[ox]^).

Sulfur is an important mineral element which is an indispensable component of sulfur-containing amino acids, including methionine and cysteine (16). Methionine plays a key role in many important biological functions, including cell proliferation, metabolism, protein synthesis, and DNA methylation (16, 17). Multiple studies have demonstrated that microorganisms have developed a complex regulatory mechanism to absorb inorganic sulfates (SO_4_^2−^) from the environment and *de novo* synthesize methionine *in vivo* (18, 19). In the natural environment, microorganisms cannot directly utilize sulfates (SO_4_^2−^) but must convert them into sulfide (S^2−^) through the sulfur uptake pathway. Figure 1 illustrates the cysteine and methionine metabolism, including the sulfur uptake pathway, methionine biosynthetic pathway A, and methionine biosynthetic pathway B (Fig. 1). The sulfur uptake pathway starts from the absorption of inorganic sulfates (SO_4_^2−^) around microorganisms catalyzed by ATP sulfurylase to form 5′-adenylyl sulfate (APS), which is subsequently converted to adenine-3′-phosphate-5′-phosphoryl sulfate (PAPS) in a reaction catalyzed by APS kinase. PAPS can be converted to sulfite (SO_3_^2−^) under the catalysis of PAPS reductase and finally converted to sulfide (S^2−^) in a reaction catalyzed by sulfite reductase. Methionine biosynthesis pathway A initially starts from the biosynthesis of cysteine from sulfide and *O*-acetyl-serine catalyzed by cysteine synthase (CS). Cysteine is metabolized to cystathionine under the catalysis of cystathionine gamma-synthase (CGS) and subsequently reduced to homocysteine in a reaction catalyzed by cystathionine beta-lyase (CBL). Methionine biosynthesis pathway B begins with the biosynthesis of *O*-acetyl-homoserine from homoserine under the catalysis of homoserine *O*-acetyltransferase (HOA), followed by the synthesis of homocysteine from *O*-acetyl-homoserine and sulfide under the catalysis of CGS, and, finally, homocysteine is metabolized to methionine under the catalysis of methionine synthase (MS) (16, 18, 20). In addition, homocysteine can also be generated through the methionine cycle, which makes it an important metabolic intermediate as it is required for the biosynthesis of sulfur-containing amino acids. In the methionine cycle, methionine can be converted to S-adenosyl methionine (SAM), which is an important methyl donor for the methylation of DNA, RNA, proteins, and lipids conducted by SAM-dependent methyltransferases (21). SAM is an important metabolic intermediate which functions as a sentinel metabolite in the control of the autophagy, eukaryotic cell cycle, and differentiation of human pluripotent stem cells (22–24). SAM can lose methyl and be converted to S-adenosyl homocysteine (SAH) and eventually to homocysteine. However, the methionine biosynthesis pathway is absent in nonruminant animals and humans. Therefore, for many antifungal drugs, such as ebelactone A and the antibiotic azoxybacillin, the methionine biosynthesis pathway has been chosen as a common target due to its absence in the animal kingdom (25–27).

In organisms, methionine is widely involved in vegetative growth, asexual development, multiple stress resistance, and pathogenicity in filamentous fungi. For instance, the deletion of *PoMet3* or *PoMet14* in Pyricularia oryzae resulted in methionine auxotrophy, defective conidiophore formation, limited infectious hyphal extension, and reduced virulence (28). In Aspergillus fumigatus, selenite sensitivity of the *laeA* mutant can be restored by overexpression of *MetR* (29). In Botrytis cinerea, *BcStr2* is involved in methionine biosynthesis, and the *BcStr2* deletion mutant exhibited reduced conidiation and virulence and increased sensitivity to osmotic and oxidative stress (30). In addition, the biosynthesis of methionine is also critical to the virulence of Magnaporthe oryzae and Fusarium graminearum (20, 31). Methionine can be transferred endogenously to cysteine, which serves an important structural role in many proteins. Cysteine is a major limiting substrate for glutathione (GSH) biosynthesis, so it is considered to be a marker for the amount of GSH (14, 32, 33). GSH serves vital functions, including antioxidant defense, modulation of immune function, regulation of cell cycle progression, and apoptosis (34–37). Numerous studies have also shown that the GSH system is essential for ROS detoxification, vegetative growth, conidiation, and virulence in many fungal pathogens, such as Candida glabrate, Candida albicans, Alternaria brassicicola, and Beauveria bassiana (38–41). Glutathione peroxidase oxidase (*GPx3*) and glutathione reductase (*Glr1*) play an important regulatory role in the glutathione cycle (oxidized glutathione [GSSG]-reduced glutathione [GSH]), which is one of the main antioxidant systems of A. alternata (14, 15). The disruption of *GPx3* or *Glr1* caused these mutants to exhibit a significant increase in ROS sensitivity and a decrease in virulence to citrus leaves (14, 15). In Alternaria alternata, YAP1 can be transported into the nucleus under oxidative stress and cooperate with the transcription factor SKN7 to regulate the downstream transcription of ROS scavenging-related genes, such as *Gpx3*, etc. (8, 9, 15).

Methionine metabolism and ROS detoxification are considered to be two independent biological processes which play crucial roles in the pathogenesis of many microbial pathogens (12, 16, 18). Previously, our research team and others have reported that the methionine biosynthesis regulator MetR plays a key role in methionine metabolism and ROS detoxification of A. alternata and many other pathogenic fungi and bacteria (16, 18, 42). Inactivation of *MetR* resulted in severe inhibition of methionine synthesis, which affected a variety of biological processes of cells (16, 43). In addition, *MetR*-disrupted mutants showed hypersensitivity to H_2_O_2_ and many ROS-generating oxidants, a phenotype that resembles those of *Yap1*, *GPx3*, *Glr1*, *Tsa1*, and *Trr1* deletion mutants (8, 16). These mutants and their phenotypes suggest a potential relationship between methionine metabolism and ROS detoxification. Transcriptome analysis and real-time PCR showed that several genes related to the cysteine and methionine metabolism pathway were significantly upregulated in *AaMetR* mutants, including *AaMetB* (cystathionine gamma-synthase), *AaMetC* (cystathionine beta-lyase), and *AaMetX* (homoserine O-acetyltransferase). Therefore, we propose that *AaMetR* may regulate methionine biosynthesis by regulating *AaMetB*, *AaMetC*, and *AaMetX* and may play an unknown role in regulating ROS tolerance (16).

In the present work, we generated *AaMetB*, *AaMetC*, and *AaMetX* deletion mutants of A. alternata and characterized the functions of *AaMetB*, *AaMetC*, and *AaMetX* by a reverse genetic strategy. Transcriptome analyses of A. alternata wild type and its derivative MetR, MetB, MetC, and MetX deletion mutants (hereafter called *ΔMetR*, *ΔMetB*, *ΔMetC*, and *ΔMetX*) were performed to explore the regulatory role of these genes. To further investigate the mechanism of MetR-regulating methionine metabolism and ROS tolerance, we also analyzed the transcriptome profiles of *AaMetR* and the wild type under vegetative growth, plant-pathogen interaction, and ROS stress. The massive gene expression data and pathways related to methionine metabolism and ROS tolerance provide us with a unique opportunity to analyze gene coexpression by weighted gene coexpression network analysis (WGCNA). Our findings not only elucidate the mechanism of methionine biosynthesis and its regulation in A. alternata but also expand and highlight the critical role of cysteine biosynthesis and its connection with glutathione-mediated ROS tolerance and virulence in pathogenic fungi and bacteria, which provides a foundation for future investigation.

## RESULTS

### Characterization and deletion of *MetB*, *MetC*, and *MetX*.

**FIG 2:**
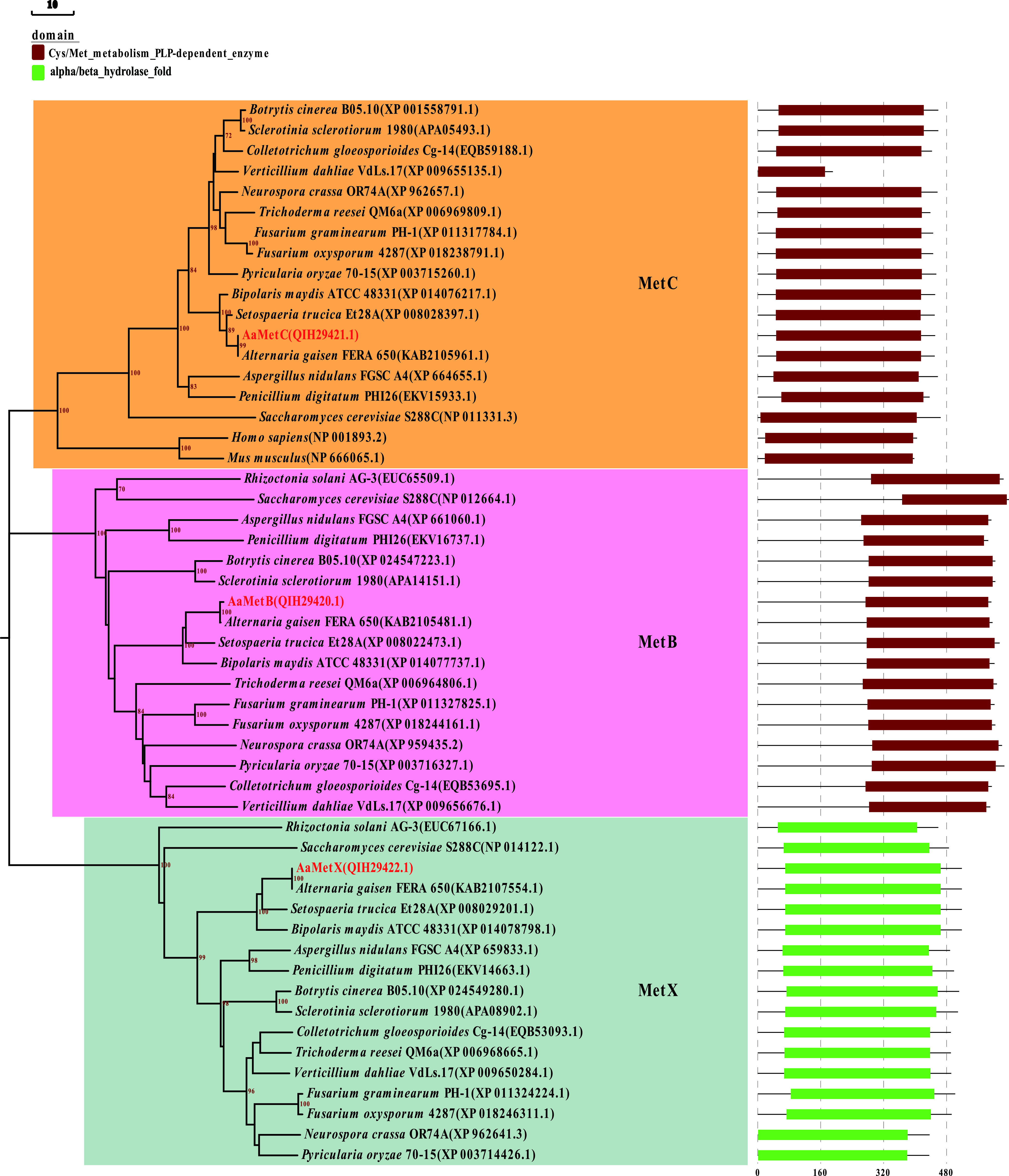
Phylogenetic analysis of cystathionine gamma-synthase (MetB), cysteine S-conjugate beta-lyase (MetC), and homoserine O-acetyltransferase (MetX) in fungi. The phylogenetic tree was constructed based on amino acid sequence alignments of the indicated protein sequences using MEGA7 by the neighbor-joining method. All positions containing gaps and missing data were eliminated. The numbers representing the percentage of replicate trees in which the associated taxa clustered together in the bootstrap test (1,000 replicates). The protein domain was predicated by InterPro (http://www.ebi.ac.uk/interpro/). The evolutionary tree and its protein domain are illustrated by EvolView v2.0 (https://evolgenius.info/evolview-v2).

The *AaMetB*, *AaMetC*, and *AaMetX* gene sequences were retrieved from the whole genome of Alternaria alternata strain Z7 (GenBank accession no. GCA_001572055.1; http://www.zjudata.com/alternaria/blast.php). The gene encoding cystathionine gamma-synthase (CGS), designated *AaMetB* (GenBank accession no. MN812273), contains a 2,157-bp open reading frame (ORF) interrupted by six introns and encodes a 594-amino-acid polypeptide which has 53.79% similarity to the CGS homolog of Pyricularia oryzae (GenBank accession no. XP_003716327.1). The gene encoding cystathionine beta-lyase (CBL), named *AaMetC* (GenBank accession no. MN812274), contains a 1,463-bp ORF interrupted by two introns of 56 bp and 51 bp and encodes a protein containing 284 amino acids that shows 77.78% amino acid identity to the CBL homolog of P. oryzae (GenBank accession no. XP_003715260.1). The gene encoding homoserine O-acetyltransferase (HOA), designated *AaMetX* (GenBank accession no. MN812275), contains a 1,828-bp ORF, interrupted by two introns of 216 bp and 52 bp, and encodes a 519-amino-acid polypeptide that shows 63.40% similarity to the HOA homolog of P. oryzae (GenBank accession no. XP_003715260.1). Protein domain analysis based on the InterPro database (https://www.ebi.ac.uk/interpro/) showed that both MetB and MetC contained the pyridoxal 5-phosphate (PLP)-dependent enzyme domains (InterPro IPR000277) for Cys/Met metabolism, while MetX contained the homoserine/serine acetyltransferase domain (InterPro IPR008220). Sequence alignment and phylogenetic analysis of the MetB, MetC, and MetX homologs of different fungi showed that yeast and filamentous fungi shared a highly conserved domain, and the protein sequence of MetX in A. alternata was most similar to that of Alternaria gaisen (Fig. 2). Interestingly, we also found MetC homologs in mice and humans, although mammals are unable to synthesize methionine *in vivo*. To determine the functions of these genes, we used a split-marker protocol to generate the gene disruption mutants. We chose the split-marker protocol because, according to previous studies, the gene disruption efficiency of this technique to produce mutants has been shown to be up to 100% for the tangerine pathotype of A. alternata (8, 12). More than 20 fungal transformants of each gene were grown on a regeneration medium. Two hygromycin-resistant transformants of each gene were randomly selected and confirmed by PCR using external primer pairs flanking the insertion site (transformants MetB1 and MetB2 for *ΔMetB*, transformants MetC1 and MetC2 for *ΔMetC*, and transformants MetX1 and MetX2 for Δ*MetX*) (Fig. S1 in the supplemental material). To compare the phenotypes of the wild-type and *AaMetR*-disrupted mutants under ROS stress, we also inoculated the *AaMetR*-disrupted mutants (transformants MetR1 and MetR2) on artificial media for morphological observation.

### Expression patterns of *MetB*, *MetC*, and *MetX* under various conditions.

**FIG 3:**
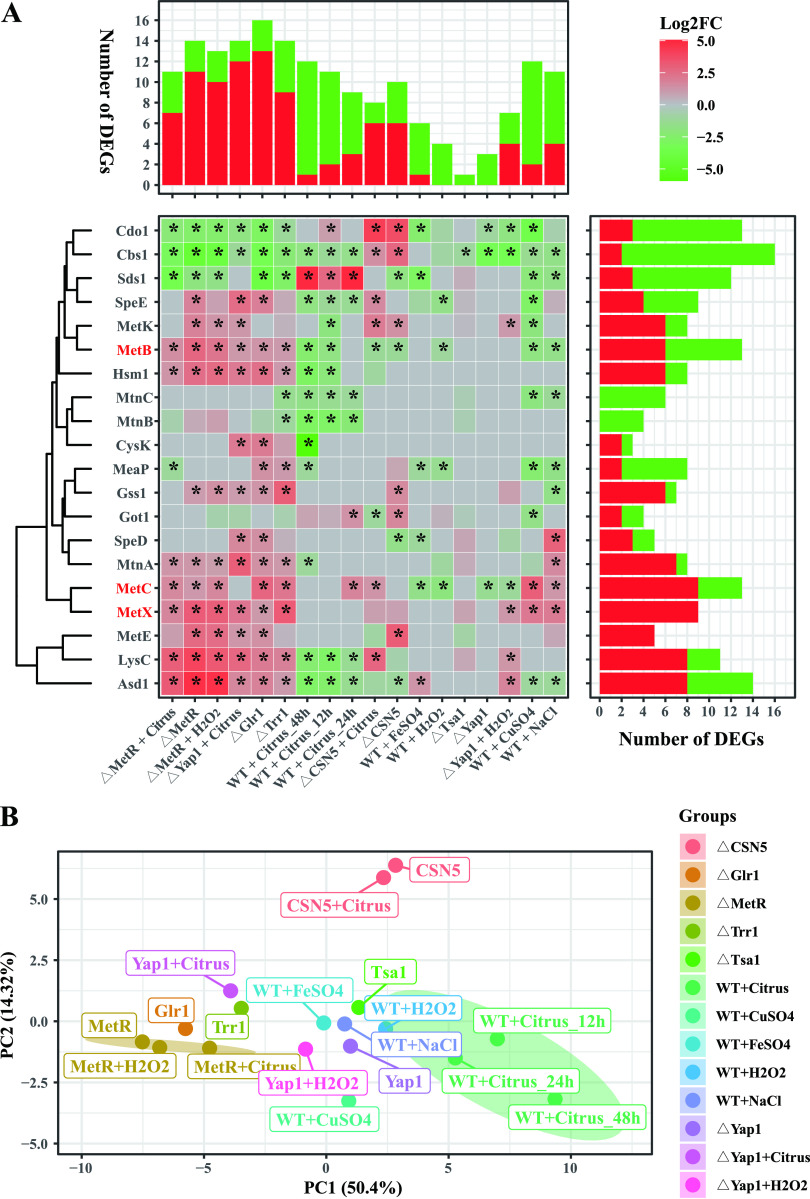
Expression profiles of cysteine and methionine metabolism-related genes. (A) Heatmap shows expression patterns of 20 genes in the Alternaria alternata wild type (WT) and its derivative mutants *ΔMetR*, *ΔCsn5*, *ΔYap1*, *ΔTsa1*, *ΔGlr1*, and *ΔTrr1* under various conditions. The tree to the left of the heatmap indicates hierarchical clustering by the genes. The bar plot above the heatmap shows the differentially expressed genes (DEGs) for each treatment. The bar plot to the right indicates the numbers of DEGs. Red, upregulation; gray, no differential expression; green, downregulation. The asterisk in the heatmap indicates a log_2_FC of ≥1.0 or ≤−1 and a false discovery rate (FDR) of ≤0.05. (B) Principal-component analysis (PCA) of the expression data of the Alternaria alternata wild type (WT) and its derivative mutants *ΔMetR*, *ΔCsn5*, *ΔYap1*, *ΔTsa1*, *ΔGlr1*, and *ΔTrr1* under various conditions. The 20 cysteine and methionine metabolism-related genes include *Asd1*, AALT_g1718, aspartate-semialdehyde dehydrogenase; *Cbs1*, AALT_g6150, cystathionine beta-synthase; *Cdo1*, AALT_g3276, cysteine dioxygenase type I; *CysK*, AALT_g7730, cysteine synthase 2; *Got1*, AALT_g5011, aspartate aminotransferase; *Gss1*, AALT_g6695, glutathione synthetase large chain; *Hsm1*, AALT_g5977, homocysteine S-methyltransferase; *LysC*, AALT_g6932, bifunctional aspartokinase/homoserine dehydrogenase; *MeaP*, AALT_g714, methylthioadenosine phosphorylase; *MetB*, AALT_g5856, cystathionine gamma-synthase; *MetC*, AALT_g4965, cystathionine beta-lyase; *MetE*, AALT_g5563, methionine synthase; *MetK*, AALT_g2537, S-adenosylmethionine synthetase; *MetX*, AALT_g1496, homoserine O-acetyltransferase; *MtnA*, AALT_g10828, methylthioribose-1-phosphate isomerase; *MtnB*, AALT_g7952, methylthioribulose-1-phosphate dehydratase; *MtnC*, AALT_g10731, 2,3-diketo-5-methylthio-1-phosphopentane phosphatase; *Sds1*, AALT_g7499, serine dehydratase; *SpeD*, AALT_g8473, S-adenosylmethionine decarboxylase; and *SpeE*, AALT_g52, spermidine synthase.

The expression patterns of *MetB*, *MetC*, *MetX*, and other methionine metabolism-related genes under 18 different conditions were analyzed. The conditions include A. alternata under oxidative stress (H_2_O_2_); infection stages at 12 h, 24 h, and 48 h postinoculation (hpi); three ionic stresses (NaCl, FeSO_4_, and CuSO_4_); two mutant stages (*ΔMetR* and *ΔCsn5*); and *ΔCsn5* inoculated with citrus leaves. The heatmap showed that the expression of *MetB*, *MetC*, *MetX*, *Hsm1*, *MtnA*, *Gss1*, *MetE*, *LysC*, and *Asd1* was upregulated (>2-fold) while the expression of *Cdo1*, *Cbs1*, and *Sds1* was downregulated (>2-fold) in the *ΔMetR* mutant. We also found that the expression of *MetB* was downregulated in the wild type supplemented with CuSO_4_ and NaCl, while the expression of *MetC* and *MetX* was upregulated in the wild type supplemented with CuSO_4_ and NaCl (Fig. 3A). Intriguingly, most of these genes, such as *MetB*, *Cbs1*, *SpeE*, *Hsm1*, *MtnC*, *MtnB*, *LysC*, and *Asd1*, were downregulated while the expression of *Sds1* was upregulated during the infection stage of A. alternata wild type. Principal-component analysis (PCA) showed that the infection stages at 12 h, 24 h, and 48 h postinoculation were clustered in one group, indicating that most of these genes shared similar expression patterns during the infection stage (Fig. 3B).

### *MetB*, *MetC*, and *MetX* are required for vegetative growth and cell development.

**FIG 4:**
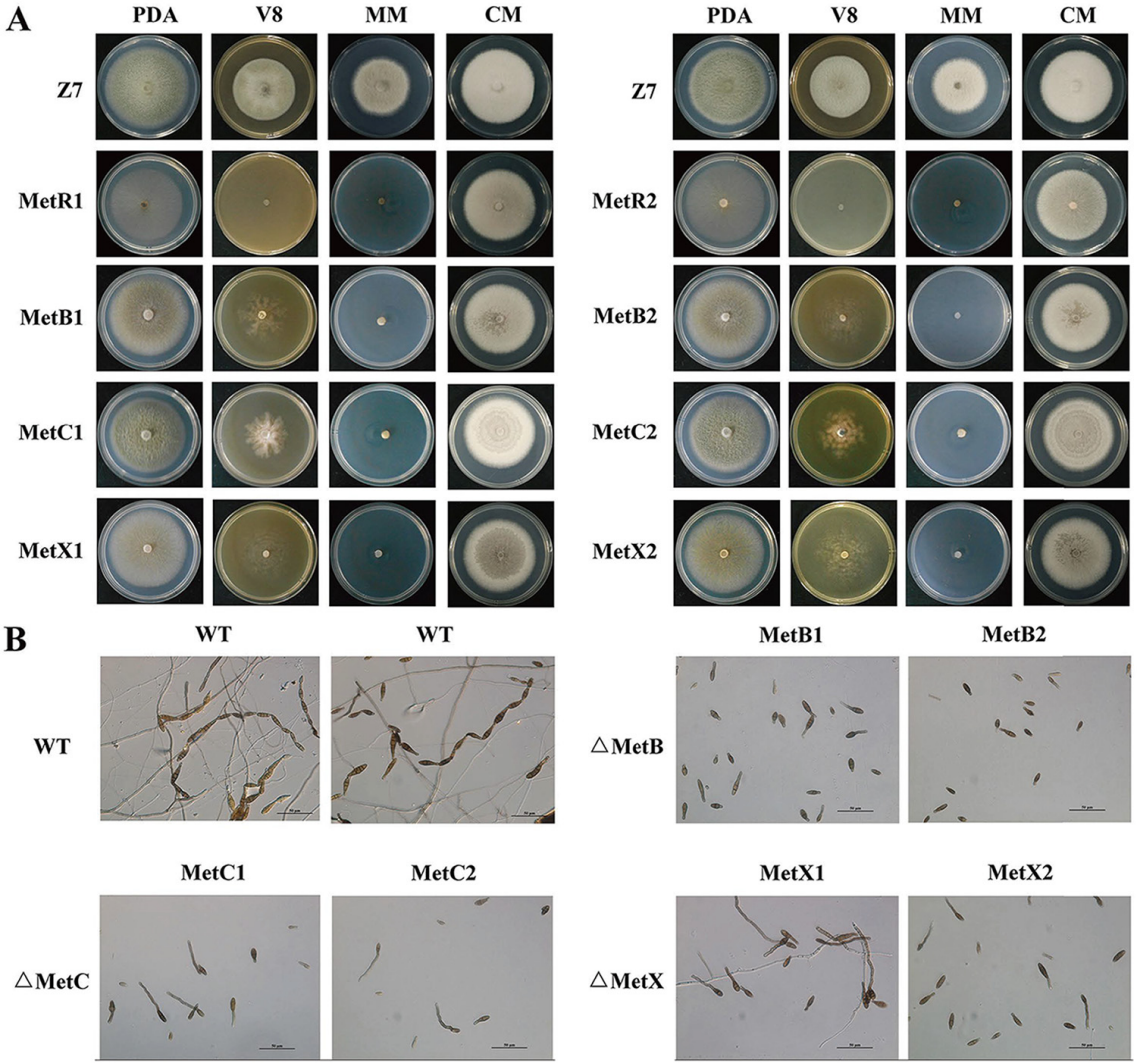
Morphological characteristics of Alternaria alternata wild type, Δ*MetR* mutant (MetR1, MetR2), Δ*MetB* mutant (MetB1, MetB2), Δ*MetC* mutant (MetC1, MetC2), and Δ*MetX* mutant (MetX1, MetX2) on artificial medium. (A) Each strain was inoculated on PDA, V8, complete medium (CM), and minimal medium (MM) and cultured at 25°C in the dark for 5 days and photographed. (B) Effect of *AaMetB*, *AaMetC*, and *AaMetX* on conidiation. The conidial production of the wild type, *ΔMetB*, *ΔMetC*, and *ΔMetX* was examined on V8 medium and cultured at 25°C in the dark for 14 days by light microscopy. All tests were repeated at least twice with three replicates of each treatment.

The colony morphology of *ΔMetB*, *ΔMetC*, and *ΔMetX* mutants is significantly different from that of the wild type on the artificial medium. On potato dextrose agar (PDA) medium, the colony of A. alternata wild-type strain Z7 was initially pale white to light brown. After 3 to 5 days, the wild-type colony turned to olivaceous to dark olive with a whitish mycelium border, and it produced abundant aerial hyphae. In contrast, the colonies of *ΔMetB*, *ΔMetC*, and *ΔMetX* mutants were consistently whitish to pale white without aerial hyphae, although the growth rates of *ΔMetB*, *ΔMetC*, and *ΔMetX* on PDA are similar to that of the wild type (Fig. 4A). Moreover, the mycelial growth of *ΔMetB*, *ΔMetC*, and *ΔMetX* mutants on V8 medium was significantly reduced, and they were completely inhibited on minimal medium (MM), indicating that *AaMetB*, *AaMetC*, and *AaMetX* are required for vegetative growth. Microscopic examination showed that the conidial yields of *ΔMetB*, *ΔMetC*, and *ΔMetX* were significantly lower than that of the wild type, indicating that the inactivation of *AaMetB*, *AaMetC*, and *AaMetX* blocked methionine metabolism and significantly affected conidiation (Fig. 4B).

### *AaMetB*, *AaMetC*, *AaMetX*, and *AaMetR* are essential for methionine and homocysteine biosynthesis.

**FIG 5:**
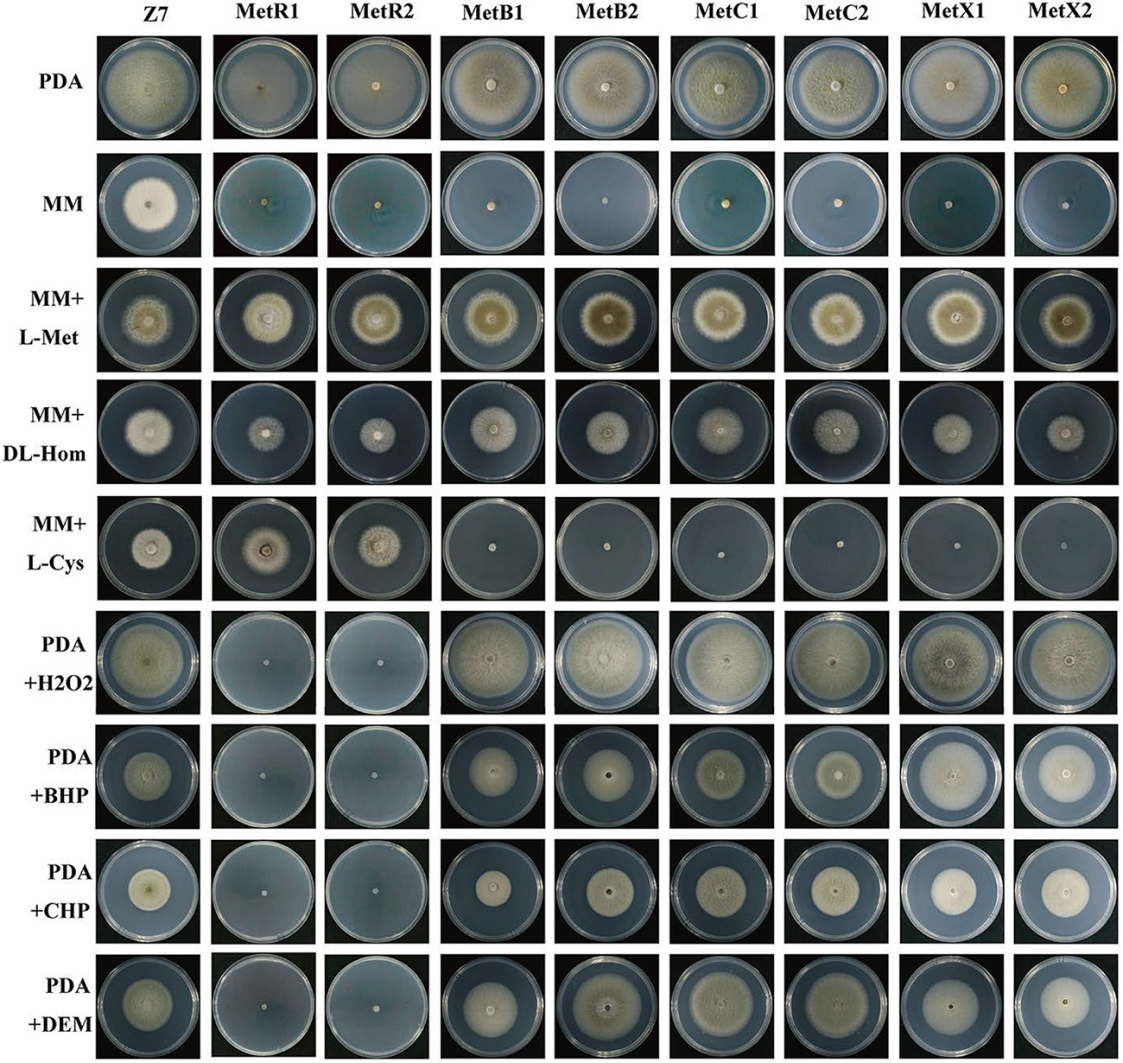
*AaMetB*, *AaMetC*, and *AaMetX* are required for methionine metabolism but not ROS tolerance, while *AaMetR* is required for cysteine metabolism and ROS tolerance. The supplementation of either methionine or homocysteine to MM restores vegetative growth and pigmentation of the Δ*MetR*, Δ*MetB*, Δ*MetC*, and Δ*MetX* strains, indicating that Δ*MetR*, Δ*MetB*, Δ*MetC*, and Δ*MetX* are methionine auxotrophs and homocysteine auxotrophs. In addition, the supplementation of cysteine to MM only restored vegetative growth of Δ*MetR*, indicating that *AaMetR* is required for cysteine metabolism. Growth of the wild-type strain, Δ*MetR*, Δ*MetB*, Δ*MetC*, and Δ*MetX* on PDA amended with either 10 mM hydrogen peroxide (H_2_O_2_), 2 mM tert-butyl-hydroperoxide (t-BHP), 1 mM cumyl hydroperoxide (CHP), or 2 mM diethyl maleate (DEM) is shown. Only the *ΔMetR* strain is hypersensitive to ROS stress. All the mutants and wild type were inoculated on media supplemented with the indicated sulfur sources or oxidants and cultured at 25°C in the dark for 5 days and photographed. All tests were repeated at least twice with three replicates of each treatment.

Previous studies have shown that the growth defect of *ΔMetR* in MM can be restored by exogenous methionine and homocysteine, which indicates *MetR* is required for methionine metabolism (16, 42). To determine whether the growth defect of each mutant on artificial media is due to the inability to synthesize methionine, *ΔMetB*, *ΔMetC*, *ΔMetX*, and *ΔMetR* were inoculated on PDA, V8, and MM supplemented with 3 mM methionine or homocysteine. After 5 days of incubation, the colony morphologies of *ΔMetB*, *ΔMetC*, *ΔMetX*, and *ΔMetR* on PDA, V8, and MM supplemented with methionine or homocysteine were similar to those of the wild type. After 2 weeks of incubation, microscopic examination of these mutants on V8 medium supplemented with methionine showed that these mutants sporulate abundantly, and the conidial morphology was similar to that of the wild type, indicating that *AaMetB*, *AaMetC*, *AaMetX*, and *AaMetR* are essential for methionine and homocysteine biosynthesis (Fig. 5 and Fig. S2). To assess production of conidia, we harvested conidia from 2-week-old cultures of each strain grown on artificial media with 5 ml sterile water. The yield of conidia from *ΔMetB*, *ΔMetC*, and *ΔMetX* mutants on V8 medium was decreased significantly compared with the wild type (Fig. S3). However, when *ΔMetB*, *ΔMetC*, and *ΔMetX* mutants were grown on V8 medium supplemented with l-methionine, these mutants produced abundant conidia. Two-week-old *ΔMetB*, *ΔMetC*, and *ΔMetX* mutants grown on V8 plates supplemented with l-methionine produced 5.4 × 10^5^ ± 0.6 × 10^5^, 7.9 × 10^5^ ± 0.7 × 10^5^, and 5.3 × 10^5^ ± 0.3 × 10^5^ conidia, respectively, levels comparable to those of wild-type strain Z7 (Fig. S3). These results indicate that the deletion of *AaMetB*, *AaMetC*, and *AaMetX* can inhibit methionine metabolism, thereby significantly reducing the production of conidia.

### *AaMetR* is essential for cysteine biosynthesis and ROS detoxification.

To determine whether the mutant phenotype can be restored by exogenous cysteine, *ΔMetR*, *ΔMetB*, *ΔMetC*, and *ΔMetX* mutants were inoculated on PDA, V8, or MM supplemented with 3 mM l-cysteine. Interestingly, only *ΔMetR* mutants exhibited a wild-type phenotype when grown on PDA, V8, or MM supplemented with cysteine (Fig. 5). In contrast, none of the *ΔMetB*, *ΔMetC*, and *ΔMetX* mutants were able to grow on MM supplemented with 3 mM l-cysteine, indicating that *AaMetR* is essential for cysteine biosynthesis (Fig. 5). The ability to detoxify ROS plays a vital role in the pathogenesis of the A. alternata tangerine pathotype. Previous studies have shown that fungal mutants lacking *AaMetR* showed hypersensitivity to H_2_O_2_ and many ROS-generating compounds, which indicates *AaMetR* is essential for ROS tolerance (16). To further investigate whether *AaMetB*, *AaMetC*, and *AaMetX* are involved in the ROS tolerance, we inoculated *ΔMetB*, *ΔMetC*, and *ΔMetX* mutants and the wild type on PDA medium supplemented with various oxidants. The *ΔMetR* mutants showed hypersensitivity to 10 mM hydrogen peroxide and other oxidants. Surprisingly, no obvious differences in ROS sensitivity were detected among *ΔMetB*, *ΔMetC*, *ΔMetX*, and wild-type fungi, indicating that *AaMetB*, *AaMetC*, and *AaMetX* are not required for ROS tolerance in A. alternata (Fig. 5).

### *ΔMetR*, but not *ΔMetB*, *ΔMetC*, or *ΔMetX*, is susceptive to chlorothalonil and salt stress.

**FIG 6:**
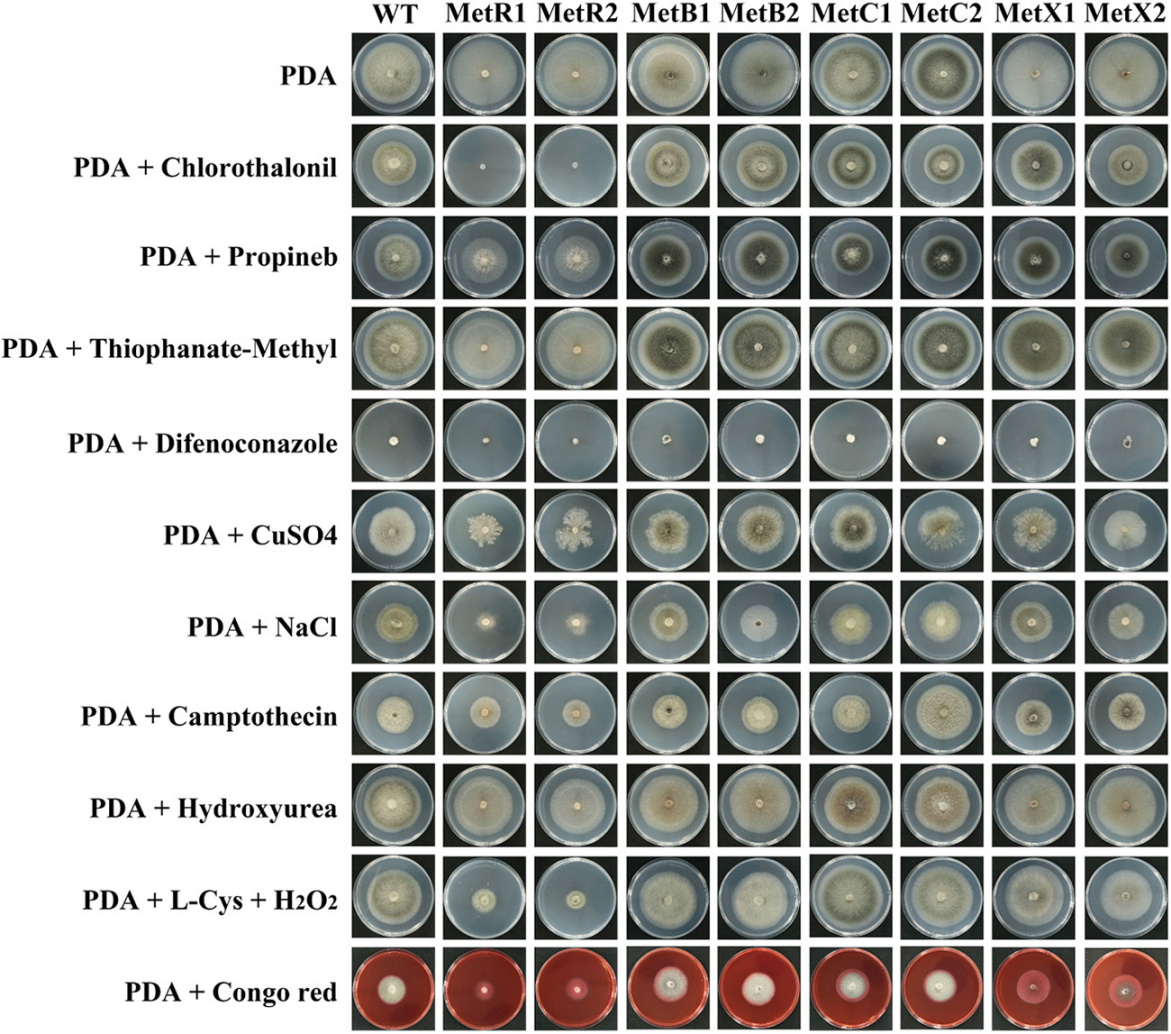
*MetR*-disrupted mutants are susceptive to salt stress and chlorothalonil. Colony characteristics of Alternaria alternata wild type, Δ*MetR*, Δ*MetB*, Δ*MetC*, and Δ*MetX* on PDA medium supplemented with different fungicides (chlorothalonil, propineb, thiophanate methyl, and difenoconazole), a copper ion stress inducer (CuSO_4_), salt stress (NaCl), cell apoptosis inducer (camptothecin and hydroxyurea), and fungal cell wall inhibitor (Congo red). The disruption of MetR led to increased sensitivity to salt stress and chlorothalonil. The supplementation of either methionine or homocysteine to MM restores vegetative growth and pigmentation of the Δ*MetR*, Δ*MetB*, Δ*MetC*, and Δ*MetX* strains, indicating that Δ*MetR*, Δ*MetB*, Δ*MetC*, and Δ*MetX* are methionine auxotrophs and homocysteine auxotrophs. In addition, the supplementation of cysteine to PDA medium amended with H_2_O_2_ partially restore vegetative growth of the Δ*MetR* strain. All the mutants and wild type were inoculated on media the indicated sulfur sources and cultured at 25°C in the dark for 5 days and photographed. All tests were repeated at least twice with three replicates of each treatment.

A fungicide is any antifungal substance that can kill or inhibit the growth of fungi and their spores. In the present work, we tested the response of these mutants to four widely used fungicides, including chlorothalonil, thiophanate methyl, propineb, and difenoconazole (Fig. 6). We chose chlorothalonil because it is a broad-spectrum protective fungicide. In theory, chlorothalonil can bind to the cysteine of glyceraldehyde-3-phosphate dehydrogenase (GAPDH), thereby inhibiting the enzyme activity and destroying the metabolism of fungal cells. Intriguingly, indoor bioassay showed that *ΔMetR* mutants, but not *ΔMetB*, *ΔMetC*, or *ΔMetX*, are hypersensitive to chlorothalonil, indicating that inactivation of *MetR* can block the cysteine biosynthesis; therefore, *ΔMetR* is more susceptible to cysteine-targeting fungicides. In addition, these mutants and the wild type are significantly inhibited on PDA medium amended with propineb and difenoconazole, but not thiophanate methyl. We also tested the sensitivity of these mutants to copper ion stress (1 mM CuSO_4_), salt stress (1 M NaCl), cell apoptosis inducer (5 μM camptothecin, 5 mM hydroxyurea), and fungal cell wall inhibitor (250 μg/ml Congo red). The *ΔMetR* mutants showed increased sensitivity to salt stress and Congo red, indicating that the inactivation of *AaMetR* partially impaired the salt stress tolerance.

### *AaMetB*, *AaMetC*, and *AaMetX* are required for fungal virulence.

**FIG 7:**
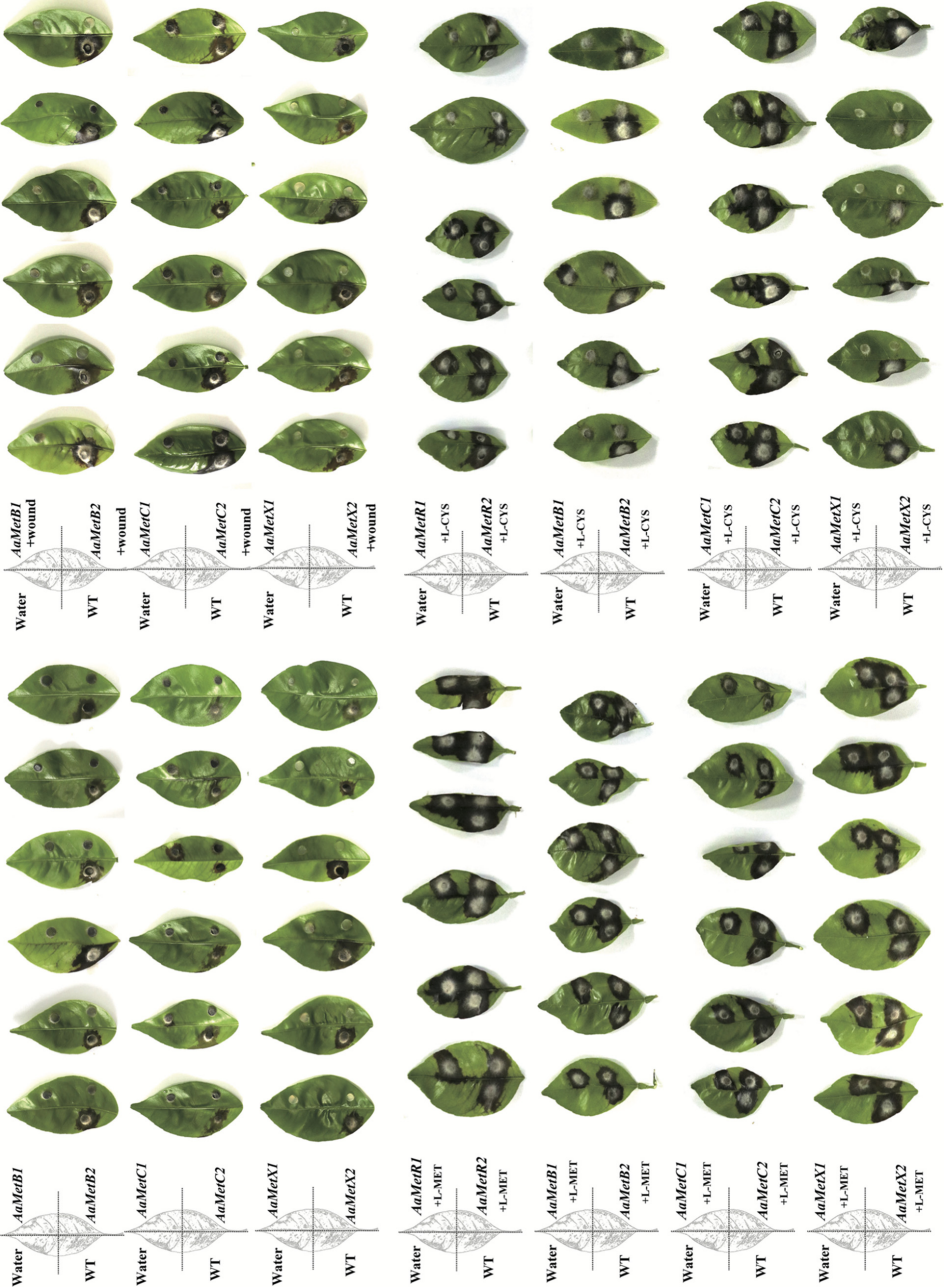
*AaMetB*, *AaMetC*, and *AaMetX* are required for Alternaria alternata pathogenicity. Pathogenicity was assayed on detached citrus leaves (Citrus reticulata Blanco) by placing a 5-mm agar plug of fungal mycelium on top of leaves. The Alternaria alternata wild-type strain Z7, Δ*MetB* mutant (MetB1, MetB2), Δ*MetC* mutant (MetC1, MetC2), and Δ*MetX* mutant (MetX1, MetX2) were grown on PDA for 3 days, and agar plugs were cut with the sterile punch of 5 mm diameter. Exogenous l-methionine compensates for the pathogenicity of Δ*MetR*, Δ*MetB*, Δ*MetC*, and Δ*MetX* mutants. The Alternaria alternata wild type, Δ*MetR* mutant (MetR1, MetR2), Δ*MetB* mutant (MetB1, MetB2), Δ*MetC* mutant (MetC1, MetC2), and Δ*MetX* mutant (MetX1, MetX2) were inoculated on PDA supplemented with l-methionine or l-cysteine for 3 days and inoculated on the detached citrus leaves for 3 days and photographed. The inoculated leaves were observed at 3 days postinoculation (dpi). Only some representative replicates are shown. All tests were repeated at least twice, and sterile water was used as control.

Previous studies have shown the MetR-regulating methionine metabolism is required for the pathogenicity of the A. alternata tangerine pathotype (16, 42). To determine whether *AaMetB*, *AaMetC*, and *AaMetX* are involved in the pathogenicity, 30 detached tangerine leaves were inoculated with 3-day-old *ΔMetB*, *ΔMetC*, and *ΔMetX* mutants and the wild-type strain. Three days after inoculation, typical symptoms, such as circular to irregular brown necrosis, were fully developed on citrus leaves inoculated with wild-type Z7. In contrast, no visible necrotic lesions were observed on citrus leaves inoculated with *ΔMetB* and *ΔMetX*. Slightly necrotic lesions were observed on detached tangerine leaves inoculated with *ΔMetC*. However, the necrotic lesions induced by *ΔMetC* were much less severe than those induced by the wild-type strain. To investigate whether the loss of pathogenicity is due to the inability of fungal mutants to penetrate host cells, the inoculation experiment was conducted on wounded citrus leaves. Three days after inoculation, wounded citrus leaves inoculated with the wild-type strain showed typical symptoms with brown spots. However, no visible symptoms were observed on the wounded citrus leaves inoculated with *ΔMetB* and *ΔMetX*, while the lesions caused by *ΔMetC* were much smaller than those induced by the wild-type strain (Fig. 7). Therefore, the inoculation assay demonstrated that the inactivation of *AaMetB* and *AaMetX* resulted in the complete loss of the virulence of A. alternata, and the disruption of *AaMetC* led to a significant decrease in its virulence.

To further investigate whether the impaired virulence is caused by methionine auxotrophy, 30 detached citrus leaves were inoculated with 3-day-old mycelial plugs of *ΔMetB*, *ΔMetC*, and *ΔMetX* grown on PDA medium supplemented with 3 mM l-methionine. Three days after inoculation, typical brown spots were observed on the detached leaves inoculated with wild-type strain Z7 as well as *ΔMetR*, *ΔMetB*, *ΔMetC*, and *ΔMetX* supplemented with 3 mM l-methionine (Fig. 7). Therefore, the reduced virulence of *ΔMetR*, *ΔMetB*, *ΔMetC*, and *ΔMetX* is due to insufficient methionine biosynthesis. We also inoculated detached tangerine leaves with 3-day-old mycelial plugs of *ΔMetR*, *ΔMetB*, *ΔMetC*, and *ΔMetX* mutants grown on PDA medium amended with 3 mM cysteine. Three days after inoculation, typical brown spot symptoms were observed on detached citrus leaves inoculated with wild-type strain Z7 as well as the *ΔMetR* mutant grown with cysteine supplementation, although the lesions induced by the *ΔMetR* mutant with cysteine supplementation were smaller than those caused by the wild-type strain (Fig. 7). Taken together, our results indicate that the loss of pathogenicity of *ΔMetB*, *ΔMetC*, and *ΔMetX* mutants is caused by the deficiency of methionine biosynthesis, which indicates that methionine biosynthesis regulated by *AaMetB*, *AaMetC*, and *AaMetX* is required for the full virulence of A. alternata tangerine pathotype.

### Data mining of transcriptome profiles.

The phenotypic characterizations of *ΔMetB*, *ΔMetC*, *ΔMetX*, and *ΔMetR* were significantly different from those of the wild-type strain. To explore the molecular mechanisms underlying the regulation of *AaMetB*, *AaMetC*, *AaMetX*, and *AaMetR*, we performed comparative transcriptomic analysis of *ΔMetB*, *ΔMetC*, *ΔMetX*, and *ΔMetR* mutants and the wild-type strain. We also analyzed the transcriptional responses of *ΔMetR* and the wild type following treatment with 10 mM H_2_O_2_ and citrus leaves. The raw transcriptome data were deposited in the Sequence Read Archive (SRA) database of the National Center for Biotechnology Information (NCBI) with the BioProject accession no. PRJNA655610. The SRA accession links for raw data, statistics of the Illumina data set, and mapping rate are shown in Table S1.

**FIG 8:**
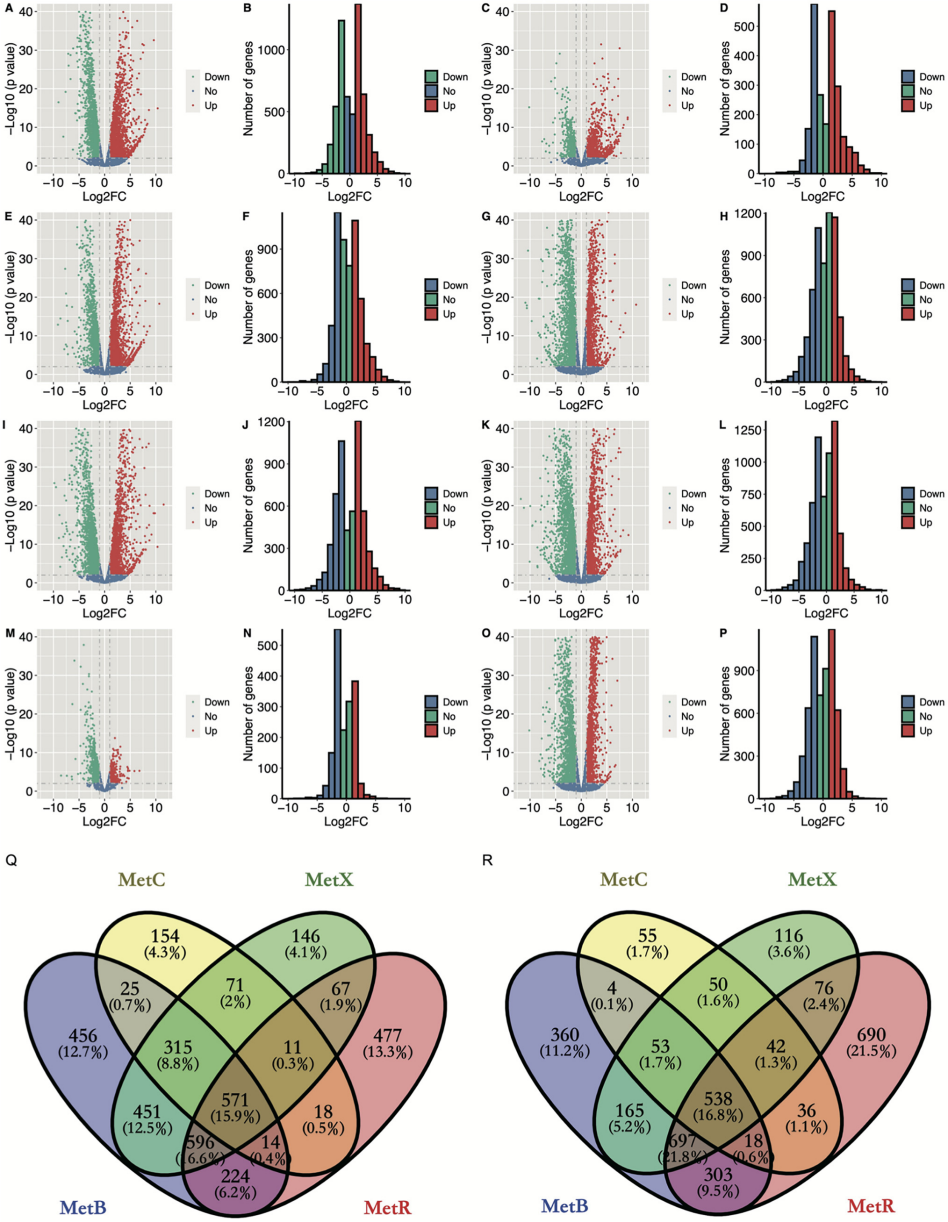
Global gene expression pattern of the mutant strains in transcriptome profiles. In the volcano plot, the *y* axis corresponds to the log_10_ mean expression value (adjusted *P* value, also known as false discovery rate [FDR]), and the *x* axis displays the log_2_FC value. Red dots represent the significantly differentially expressed transcripts (FDR < 0.05) which have been upregulated in the indicated strains compared to the wild type. Green dots represent the significantly differentially expressed transcripts (FDR < 0.05) which have been downregulated in the indicated strains compared to the wild type. Blue dots represent the transcripts whose expression levels did not reach statistical significance (FDR > 0.05) or the absolute value of log_2_FC value is smaller than 1. (A and B) Volcano plot (A) and histogram plot (B) of the Δ*MetB* mutant’s gene expression pattern. (C and D) Volcano plot (C) and histogram plot (D) of the Δ*MetC* mutant’s gene expression pattern. (E and F) Volcano plot (E) and histogram plot (F) of the Δ*MetX* mutant’s gene expression pattern. (G and H) Volcano plot (G) and histogram plot (H) of the Δ*MetR* mutant’s gene expression pattern. (I and J) Volcano plot (I) and histogram plot (J) of the Δ*MetR* mutant supplemented with H_2_O_2_. (K and L) Volcano plot (K) and histogram plot (L) of the Δ*MetR* mutant inoculated with citrus leaves. (M and N) Volcano plot (M) and histogram plot (N) of the wild type supplemented with H_2_O_2_. (O and P) Volcano plot (O) and histogram plot (P) of the wild type inoculated with citrus leaves. (Q) Venn diagram describing overlaps of upregulated genes among the mutant strains *ΔMetB*, *ΔMetC*, *ΔMetX*, and *ΔMetR* in PDB medium. (R) Venn plot describing overlaps of downregulated genes among the mutant strains *ΔMetB*, *ΔMetC*, *ΔMetX*, and *ΔMetR* in PDB medium.

The differentially expressed genes (DEGs) were identified with the threshold of an absolute value of log_2_(fold change) (FC) of ≥1 and false discovery rate (FDR) of ≤0.05 (Fig. 8). In total, 4,790 genes were different in *ΔMetB* and the wild-type strain, comprising 2,652 upregulated genes and 2,138 downregulated genes (Fig. 8A and B). For the *ΔMetC* mutant, 1,975 genes were differentially expressed, including 1,179 upregulated genes and 796 downregulated genes (Fig. 8C and D). For the *ΔMetX* mutant, 3,965 genes were differentially expressed, including 2,228 upregulated genes and 1,737 downregulated genes (Fig. 8E and F). For the *ΔMetR* mutant, 4,378 genes were differentially expressed, including 1,978 upregulated genes and 2,400 downregulated genes (Fig. 8G and H). For the *ΔMetR* mutant treated with H_2_O_2_, 4,730 genes were differentially expressed, including 2,384 upregulated genes and 2,346 downregulated genes (Fig. 8I and J). For the *ΔMetR* mutant inoculated with citrus leaves, 4,749 genes were differentially expressed, including 2,114 upregulated genes and 2,635 downregulated genes (Fig. 8K and L). For the *ΔMetR* mutant, 1,172 genes were differentially expressed, including 250 upregulated genes and 922 downregulated genes (Fig. 8G and H). For the wild type treated with H_2_O_2_, 1,222 genes were differentially expressed, including 456 upregulated genes and 766 downregulated genes (Fig. 8M and N). For the wild type inoculated with citrus leaves, 4,382 genes were differentially expressed, including 2,098 upregulated genes and 2,284 downregulated genes (Fig. 8O and P). Twelve genes were randomly selected for quantitative reverse transcription-PCR (qRT-PCR) to verify the results of the transcriptome study. Although the fold changes are slightly different, the gene expression pattern in qRT-PCR is consistent with the gene expression pattern in the transcriptome data (Fig. S4).

Comparative transcriptome analysis was performed to systematically define the union and intersection of DEGs between different strains and treatments. The Venn plot showed that there were 571 upregulated genes and 538 downregulated genes in *ΔMetB*, *ΔMetC*, *ΔMetX*, and *ΔMetR*, indicating that the inactivation of *AaMetB*, *AaMetC*, *AaMetX*, and *AaMetR* led to methionine auxotrophy that elicited a common transcriptional response (Fig. 8Q and R). Circos plots displayed the genomic location of genes differentially expressed in *ΔMetB*, *ΔMetC*, *ΔMetX*, and *ΔMetR* mutants, as well as the positions, lengths, and expression levels of genes involved in secondary metabolite biosynthesis (Fig. S5 to S8). Interestingly, we found that most of these genes on the conditionally dispensable chromosome (CDC) are upregulated in *ΔMetB*, *ΔMetC*, and *ΔMetX*, but downregulated in *ΔMetR*, indicating that the regulatory patterns of MetB, MetC, MetX, and MetR on the genes of CDC are different.

### Deletion of *AaMetB*, *AaMetC*, *AaMetX*, and *AaMetR* widely affects the expression of secondary metabolite gene clusters.

A. alternata can produce many secondary metabolites (SM) that enable them to adapt to various ecological environments. To explore the relationship of secondary metabolites and pathogenicity mediated by *AaMetB*, *AaMetC*, *AaMetX*, and *AaMetR*, we examined the transcription responses of 30 biosynthetic gene clusters in A. alternata predicted by antiSMASH 5.0. The levels of gene expression of cluster 2 type 1 polyketide synthase (T1PKS) (alternariol), cluster 7 T1PKS, cluster 18 nonribosomal peptide synthetase (NRPS), cluster 23 NRPS-like, cluster 28 T1PKS (mellein), and cluster 29 NRPS (ACT toxin I) were strongly upregulated while the transcript levels of cluster 5 T1PKS (alternapyrone) were significantly downregulated in *ΔMetB*, *ΔMetC*, and *ΔMetX* (Fig. S9 to S11). In *ΔMetR*, the expression levels of cluster 6 NRPS (dimethylcoprogen), cluster 19 NRPS-like (phomopsins), and cluster 20 NRPS-like were strongly upregulated while the expression level of cluster 5 T1PKS (alternapyrone) was significantly downregulated (Fig. S12 and Fig. S13). In contrast, only a few secondary metabolite genes in the wild type were affected under oxidative stress, indicating that H_2_O_2_ treatment had no significant effect on the gene expression of secondary metabolism. After inoculation of the wild type with citrus leaves, the expression of several genes in cluster 5 T1PKS (alternapyrone) and cluster 16 NRPS-like were significantly downregulated, indicating that the expression of cluster 5 T1PKS (alternapyrone) and cluster 16 NRPS-like decreased during the host-pathogen interaction. The host-selective ACT toxin regulated by the ACT toxin biosynthesis gene cluster is essential for the pathogenicity of A. alternata tangerine pathotype. Interestingly, we found that although the virulence of *ΔMetB*, *ΔMetC*, *ΔMetX*, and *ΔMetR* is significantly reduced or completely lost, most of these genes in the ACT toxin biosynthesis gene cluster were upregulated in *ΔMetB*, *ΔMetC*, and *ΔMetX* but downregulated in *ΔMetR*, which indicates that inactivation of these genes can strongly affect the biosynthesis of ACT toxin in the tangerine pathotype of A. alternata (Fig. S9 to Fig. S13).

### Deletion of *AaMetB*, *AaMetC*, *AaMetX*, and *AaMetR* broadly affects many critical metabolic pathways.

**FIG 9:**
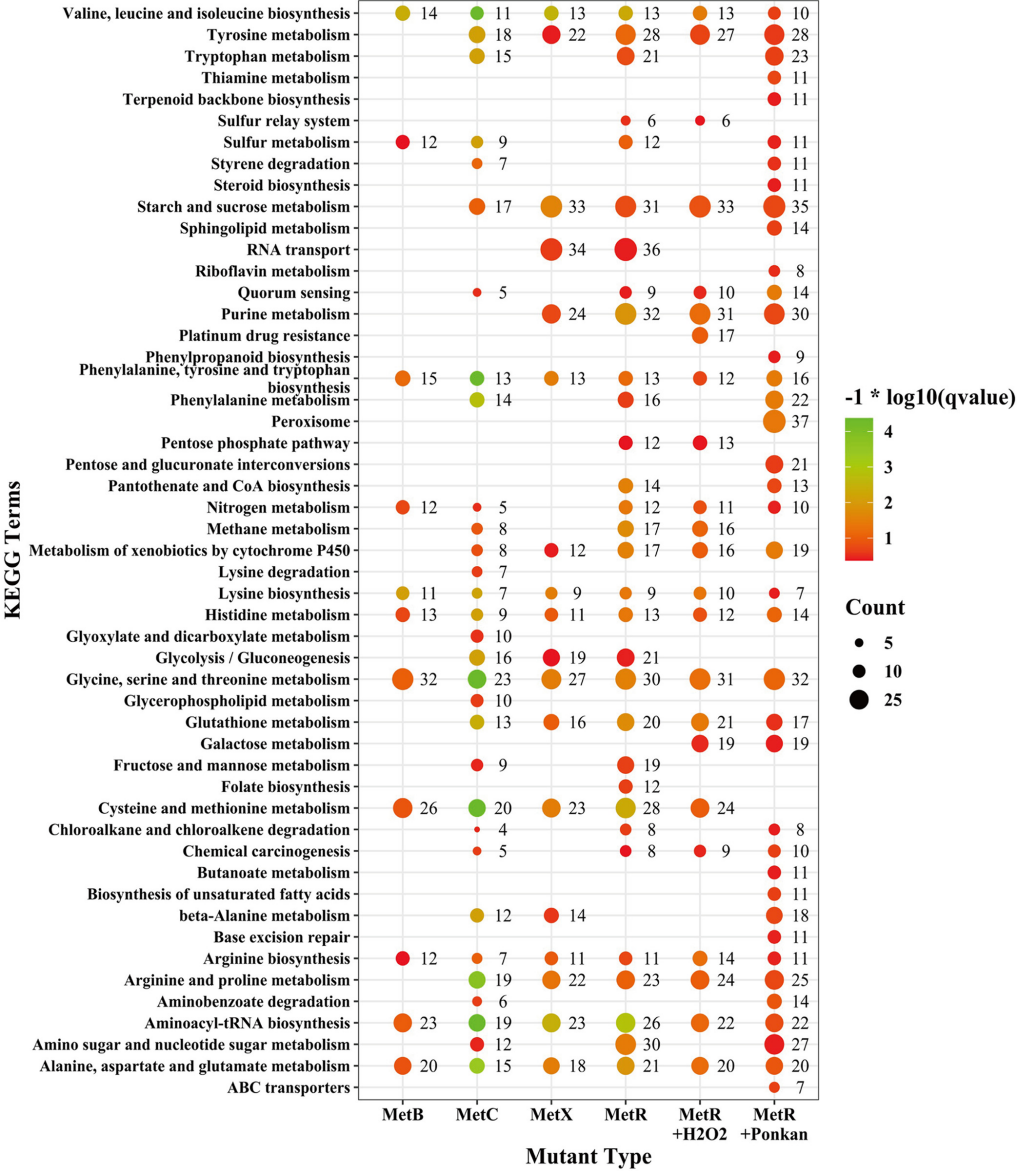
Comparative KEGG pathway analysis among *ΔMetR*, *ΔMetB*, *ΔMetC*, *ΔMetX*, and *ΔMetR* supplemented with H_2_O_2_ and *ΔMetR* inoculated with citrus leaves. Count represents the numbers of differentially expressed genes annotated in the pathway term. The *q* value is the adjusted *P* value.

Comparative gene ontology (GO) and KEGG pathway analysis were performed to explore the function and regulatory role of DEGs in each mutant. After assigning DEGs to the GO category, most DEGs belong to “protein binding,” “oxidoreductase activity,” “DNA binding,” “zinc ion binding,” “ATP binding,” “transmembrane transport,” “carbohydrate metabolic process,” and “integral component of membrane” (Fig. S14). KEGG pathway analysis showed that the inactivation of *AaMetB*, *AaMetC*, and *AaMetX* extensively affects the gene expression in several critical metabolic pathways, including the biosynthesis of valine, leucine, and isoleucine; phenylalanine, tyrosine, and tryptophan biosynthesis; alanine, aspartate, and glutamate metabolism; and glycine, serine, and threonine metabolism (Fig. 9). In particular, we found that 22, 15, and 21 DEGs enriched in cysteine and methionine metabolism were significantly upregulated in *ΔMetB*, *ΔMetC*, and *ΔMetX*, respectively. We also found that inactivation of *AaMetR* affects the expression of 20 genes enriched in glutathione metabolism, which is an important pathway related to ROS tolerance (Fig. S15). In addition, the inactivation of *AaMetB*, *AaMetC*, and *AaMetX* broadly influenced the expression of 139, 55, and 110 transcription factor genes, respectively, indicating that the inactivation of *AaMetB*, *AaMetC*, and *AaMetX* may affect the expression of myriad downstream genes.

### Inactivation of *AaMetR* affects several critical genes related to ROS tolerance.

**FIG 10:**
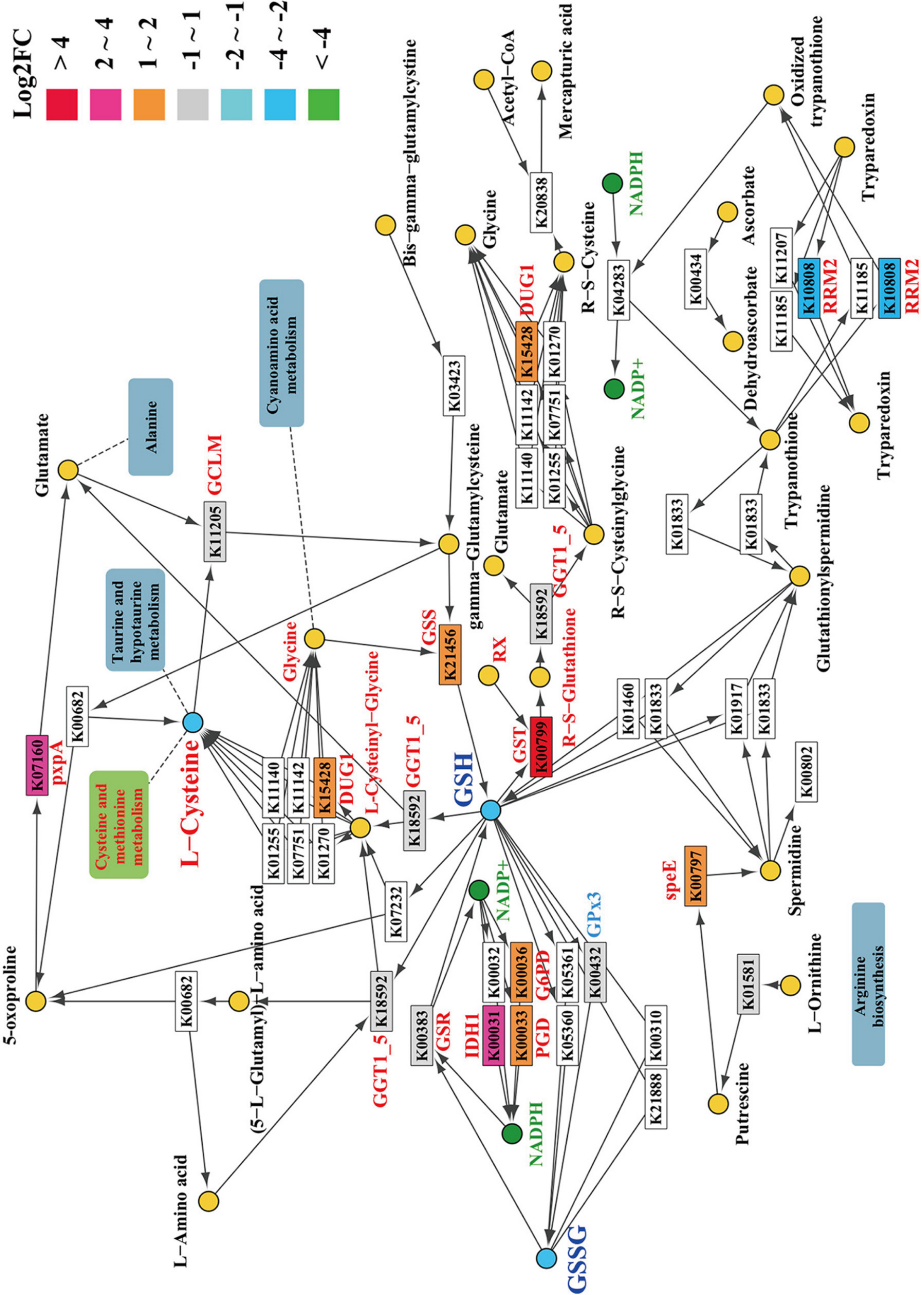
The KEGG pathway figure shows differential expression of genes in the glutathione metabolism of the *ΔMetR* mutant. The genes from transcriptome data that were differentially expressed by the *ΔMetR* mutant and the wild type were mapped against glutathione metabolism. The different colors of the squares represent differential gene expression in the *ΔMetR* mutant compared to the wild type. The square without color indicates the gene that is not present in Alternaria alternata.

The transcriptional factor MetR is a critical regulator affecting the ROS tolerance and pathogenicity of A. alternata (16). In contrast, phenotypic analysis showed that methionine auxotrophs such as *ΔMetB*, *ΔMetC*, and *ΔMetX* are not sensitive to ROS stresses, indicating that *AaMetB*, *AaMetC*, and *AaMetX* are not essential for ROS tolerance. To better understand the regulatory role of AaMetR under ROS stress, we performed a comparative analysis of *ΔMetR* and wild-type fungi under conditions of normal growth, ROS stress, and citrus leaf inoculation. We found that the inactivation of *AaMetR* significantly affected 20 genes in the glutathione metabolic pathway (Fig. S15 and Fig. 10). Following treatment with 3 mM H_2_O_2_, 6 genes and 21 genes related to glutathione metabolism were differentially expressed in the wild type and *ΔMetR*, respectively. Glutathione is the major antioxidant of the glutaredoxin system, which is regulated by glutathione peroxidase 3 (GPx3) and glutathione-disulfide reductase (Glr1). Therefore, MetR regulates the biosynthesis of cysteine, which is the biosynthetic precursor of methionine and glutathione. As mentioned above, the inactivation of *AaMetR* can block the biosynthesis of cysteine, which renders the mutant unable to synthesize glutathione to detoxify ROS and also causes the mutant to be unable to synthesize methionine to participate in various metabolisms. We also found that 37 genes enriched in the peroxisome were differentially expressed in *ΔMetR* inoculated with citrus leaves, indicating that multiple peroxisome-related genes were widely affected in the *ΔMetR* mutant during plant-pathogen interaction. Weighted gene coexpression network analysis (WGCNA) demonstrated that there is extensive gene coexpression among cysteine and methionine metabolism, tryptophan metabolism, tyrosine metabolism, and peroxisome, indicating that there are potential connections between peroxisome and multiple metabolic pathways (Fig. S16).

## DISCUSSION

The bZIP transcription factor MetR plays an important role in the regulation of methionine metabolism, ROS tolerance, and pathogenicity in Magnaporthe oryzae (42), Aspergillus fumigatus (29), A. alternata (16), and Cryptococcus neoformans (18). Interestingly, a recent study found that a LysR-type transcription factor, MetR, in Serratia marcescens, which has a conserved protein domain commonly present in bacteria, plays a critical role in methionine biosynthesis, ROS tolerance, prodigiosin production, cell motility, heat tolerance, and exopolysaccharide synthesis (43). Previously, we found that the cystathionine gamma-synthase gene (*AaMetB*), cystathionine beta-lyase gene (*AaMetC*), and homoserine O-acetyltransferase gene (*AaMetX*) were upregulated in the *AaMetR*-disrupted mutant. In this work, we characterized the biological function of *AaMetB*, *AaMetC*, and *AaMetX* in the tangerine pathotype of A. alternata, which is an important necrotroph that causes *Alternaria* brown spot of citrus worldwide.

Nutritional adaptation plays a vital role in the survival of many filamentous fungi, which enables them to occupy diverse ecological niches in the environment, from the icy tundra to the tropical rainforests to the deserts (18). The ability to take up and recycle multiple nutrients confers advantages to filamentous fungi for their survival and proliferation under different conditions. Through a targeted gene disruption approach, we found that the *AaMetB*, *AaMetC*, and *AaMetX* are required for the vegetative growth, conidiation, and pathogenicity of A. alternata. Fungal mutants lacking either *AaMetB*, *AaMetC*, or *AaMetX* displayed developmental defects such as reduced aerial hyphae and conidia and the inability to grow on minimum medium. However, the defects in cell growth and development of these mutants can be restored by exogenous methionine or homocysteine but not cysteine, which indicates that *AaMetB*, *AaMetC*, and *AaMetX* mutants are typical methionine auxotrophs rather than cysteine auxotrophs. These experiments further proved that *AaMetB*, *AaMetC*, and *AaMetX* play an important role in the growth and development of A. alternata.

Our pathogenicity assays showed that the *ΔMetB* and *ΔMetX* mutants could not induce necrotic lesions on the detached citrus leaves, while the pathogenicity of *ΔMetC* was greatly reduced, indicating that *AaMetB*, *AaMetC*, and *AaMetX* are required for wild-type levels of pathogenicity. The impaired pathogenicity of these mutants on citrus leaves can be restored by exogenous methionine but not cysteine, which further demonstrates that *AaMetB*, *AaMetC*, and *AaMetX* mutants are methionine auxotrophs. Previously, host-selective ACT toxin and ROS detoxification were considered to be two critical determinants of pathogenicity of A. alternata (7, 8, 12). In another study, *AaSte12* was found to be involved in the pathogenesis of A. alternata, regulating the formation of conidia and the production of cell wall-degrading enzymes (CWDEs) (44). In the present study, we found that blocking the methionine biosynthesis in A. alternata renders the mutants avirulent or less virulent and causes them to produce fewer conidia, which emphasized that methionine is required for conidiation and wild-type levels of pathogenicity. In Magnaporthe oryzae, the cystathionine gamma-synthase encoded by *MoMetB* is essential for methionine metabolism and virulence (45). In F. graminearum, fungal mutants lacking *FgMetB* exhibited decreased virulence to wheat, which is consistent with low levels of deoxynivalenol (DON) production in wheat kernels (20). In Botrytis cinerea, mutants lacking *BcMetB* exhibited significantly decreased virulence on host plant tissues (30). Consistent with our previous findings, this study demonstrated the important role of *AaMetB*, *AaMetC*, and *AaMetX* in the full pathogenicity of A. alternata, indicating that *AaMetB*, *AaMetC*, and *AaMetX*, involved in methionine biosynthesis, are critical factors related to the virulence of fungal pathogens.

The transcriptomes of *ΔMetB*, *ΔMetC*, *ΔMetX*, *ΔMetR*, and wild type were analyzed to explore the regulatory role of these genes in cysteine and methionine metabolism. The results showed that the inactivation of *AaMetB*, *AaMetC*, and *AaMetX* blocked methionine metabolism, thus widely affecting the expression of many amino acid metabolism-related genes. To further investigate the transcriptional response of *ΔMetR* and the wild type under ROS stress and plant-pathogen interaction, we also performed transcriptome profiles of *ΔMetR* and the wild type inoculated with H_2_O_2_ and citrus leaves. In our transcriptome data, we found that *AaMetB*, *AaMetC*, and *AaMetX* can block methionine metabolism and extensively affect the gene expression of multiple secondary metabolite biosynthetic gene clusters. Fungal secondary metabolites are organic metabolites produced by secondary metabolite gene clusters, which enable fungi to occupy a unique ecological niche in the environment (46, 47). Among the 30 secondary metabolite gene clusters predicted by antiSMASH, the alternapyrone biosynthesis gene cluster was significantly downregulated in *AaMetB*, *AaMetC*, *AaMetX*, and *AaMetR* deletion mutants, as well as the wild type inoculated on citrus, indicating that there are potential links between alternapyrone biosynthesis and methionine as well as host-pathogen interactions. In the *ΔMetB*, *ΔMetC*, and *ΔMetX* mutants, the gene expression of the ACT toxin biosynthesis gene cluster was strongly upregulated. In contrast, the transcription level of the ACT toxin biosynthesis gene cluster in *ΔMetR* was strongly downregulated, indicating that inactivation of these genes can block the cysteine and methionine biosynthesis pathway, thereby affecting the expression of the ACT toxin biosynthesis gene cluster.

The ability to detoxify host-generated ROS is critical to the pathogenicity of numerous plant pathogens. Previous studies have also shown that the inactivation of antioxidant genes such as *AaTsa1*, *AaTrr1*, *AaGlr1*, *AaGPx3*, *AaAp1*, *AaSkn7*, and *AaHog1* in A. alternata renders these mutant strains more susceptible to ROS stress and less virulent to host plants. Our previous studies have shown that the transcriptional factor AaMetR contributes to the oxidative stress tolerance and virulence of A. alternata, which suggests there may be a potential link between methionine metabolism and ROS tolerance (16, 18, 42). With this in mind, we evaluated the response of the *ΔMetR*, *ΔMetB*, *ΔMetC*, and *ΔMetX* mutants to various oxidants, including H_2_O_2_, butyl-hydroperoxide (BHP), cumyl hydroperoxide (CHP), and diethyl maleate (DEM). Surprisingly, the *AaMetR*-disrupted mutants are hypersensitive to these oxidants, while *AaMetB*, *AaMetC*, and *AaMetX* deletion mutants are not sensitive to ROS stress. We also found that inactivation of *AaMetR* extensively affects the expression of many genes associated with cysteine and methionine metabolism and glutathione metabolism. Therefore, we speculate that the inactivation of *AaMetR* can block the biosynthesis of cysteine, which is an indispensable substrate for glutathione biosynthesis. Moreover, glutathione is the major substance in the glutaredoxin system and is essential for ROS tolerance, while methionine is an important amino acid for vegetative growth and pathogenicity. The impaired resistance of *ΔMetR* to ROS stress can be partially restored by exogenous cysteine, which further demonstrates that inactivation of MetR can block the biosynthesis of cysteine, thereby affecting ROS resistance. Laboratory bioassays showed that only *ΔMetR* mutants are susceptible to the fungicide chlorothalonil, which can combine with the cysteine of GADPH, further confirming the important role of *AaMetR* in cysteine metabolism. Therefore, these results indicate that *AaMetB*, *AaMetC*, and *AaMetX* are required for the biosynthesis of methionine, which is essential for fungal development and pathogenicity of A. alternata. In contrast, the bZIP transcriptional factor MetR is required for the biosynthesis of cysteine, which is an indispensable substrate in the biosynthesis of methionine and glutathione and is essential for ROS tolerance, fungal development, and pathogenesis in A. alternata.

Taken together, the present study suggests the presence of a mechanism whereby MerR regulating cysteine biosynthesis plays an important role in the methionine metabolism catalyzed by MetB, MetC, and MetX and glutathione-mediated ROS detoxification catalyzed by GPx3 and GLR1. Inactivation of *AaMetR* can block the cysteine biosynthesis pathway, making the mutants unable to provide sufficient cysteine to further synthesize methionine and glutathione. Methionine is an important sulfur-containing amino acid which is related to fungal growth, cell development, and pathogenicity. Glutathione is an essential antioxidant that can catalyze the detoxification of ROS in the glutaredoxin system. Due to the shortage of cysteine in *ΔMetR*, genes related to methionine metabolism (such as *AaMetB*, *AaMetC*, and *AaMetX*) were significantly upregulated for synthesis of sufficient methionine to maintain cell growth and development of the fungi. The inactivation of *AaMetB*, *AaMetC*, and *AaMetX* can block methionine biosynthesis and cause methionine auxotrophy. This work highlights the critical roles of sulfur-containing amino acids such as cysteine, homocysteine, and methionine in fungal growth, conidiation, and pathogenicity of phytopathogens. Furthermore, cysteine plays an important role in ROS detoxification in filamentous fungi due to its being an irreplaceable substrate in the biosynthesis of glutathione. These findings not only emphasize the key role of methionine metabolism regulated by MetB, MetC, and MetX in fungal growth and conidiation and its connection with the virulence of plant-pathogenic fungi but also underline the indispensable role of MetR in cysteine and methionine metabolism, as well as ROS detoxification, in filamentous fungi and provides a foundation for future research.

## MATERIALS AND METHODS

### Fungal strains and culture conditions.

The A. alternata strain Z7 was isolated from a diseased citrus leaf sampled in Wenzhou, Zhejiang Province, China, and served as the wild type for the mutagenesis experiments (48, 49). Fungal mutant *ΔMetR*, whose bZIP transcription factor MetR is impaired, exhibits a typical phenotype of methionine auxotrophy and was generated in a previous study (16). Fungal strains were cultured on potato dextrose agar (PDA) medium (200 g potato, 20 g glucose, and 20 g agar per liter of purified water), V8 medium (200 ml V8 broth, 3 g CaCO_3_, and 20 g agar per liter of purified water), minimal medium (MM; 0.5 g KCl, 2 g NaNO_3_, 1 g KH_2_PO_4_, 0.5 g MgSO_4_·7H_2_O, 0.01 g FeSO_4_, 10 g sucrose, 200 μl trace elements, and 20 g agar per liter of purified water), and complete medium (CM; MM with 1 g yeast extract, 1 g casein hydrolysate, and 2 g peptone per liter) at 25°C to evaluate their growth and colony characteristics. The trace element solution consists of 5 g of ZnSO_4_, 5 g of citric acid, 0.25 g of CuSO_4_·5H_2_O, and 1 g of (NH_4_)_2_Fe(SO_4_)_2_·6H_2_O per 100 ml of purified water. PDB medium includes 200 g potato, 20 g glucose, and 1 liter of purified water. Regeneration medium contains 1 M (342 g) sucrose, 1 g of peptone, 1 g of yeast extract, and 1 liter of purified water. STC buffer contains 1.2 M sorbitol (109.32 g), 5 ml of 1 M Tris, 5 ml of 1 M CaCl_2_, and 500 ml of purified water. The osmotic buffer contains 70 g of NaCl, 20 ml of 1.0 M CaCl_2_, 25 ml of 0.4 M Na_2_HPO_4_, and 1 liter of purified water.

### Expression patterns of methionine metabolism-related genes under various conditions.

To identify the expression patterns of methionine metabolism-related genes under various conditions, the expression of 20 genes, including *MetB*, *MetC*, *MetX*, *Asd1*, *Cbs1*, *Cdo1*, *CysK*, *Got1*, *Gss1*, *Hsm1*, *LysC*, *MeaP*, *MetE*, *MetK*, *MtnA*, *MtnB*, *MtnC*, *Sds1*, *SpeD*, and *SpeE*, was measured under conditions of oxidative stress (10 mM H_2_O_2_), salt stress (1 M NaCl), and metal ion stress (3 mM FeSO_4_, 1 mM CuSO_4_) in a pathogen-infectious period (12 h, 24 h, 36 h), and different mutants (*ΔMetR*, *ΔGlr1*, *ΔTsa1*, *ΔTrr1*, and *ΔCsn5*) were analyzed. The gene expression heatmap was visualized by R ggplot2 package. The asterisk in the heatmap indicates an absolute value of log_2_FC of ≥1 and a false discovery rate (FDR) of ≤0.05. The principal-component analysis (PCA) was visualized by the R ggplot2 package.

### Identification and phylogenetic analysis of MetB, MetC, and MetX.

The homologs of *AaMetB*, *AaMetC*, and *AaMetX* in A. alternata strain Z7 were identified by KEGG enrichment analysis, comparing the transcriptome sequencing (RNA-Seq) data of the *ΔMetR* mutant with that of the wild type from the previous study (16). MetB, MetC, and MetX homologs from different fungi used in the phylogenetic analysis were identified using BLAST and downloaded from the GenBank sequence database of NCBI. The conserved domains of each protein were identified in the InterPro protein database (https://www.ebi.ac.uk/interpro/). Multiple protein sequence alignment was carried out using ClustalW. Phylogenetic trees were constructed using the neighbor-joining (NJ) method with 1,000 bootstrap replicates in MEGA7. The phylogenetic tree with protein domain annotation was visualized in EvolView v2 (https://www.evolgenius.info/evolview/).

### Construction of *AaMetB*, *AaMetC*, and *AaMetX* deletion mutants.

The *AaMetB*, *AaMetC*, and *AaMetX* gene-disrupted mutants were generated by homologous recombination and protoplast transformation as previously described (16). In brief, two fragments (HY/g and h/YG) containing a partial hygromycin resistance gene (HYG) cassette driven by a *trpC* gene promoter were amplified by PCR from the pTFCM vector with the primer pairs M14R/Hyg3 and M14F/Hyg4, respectively. The upstream and downstream flanking regions of the *AaMetB*, *AaMetC*, and *AaMetX* genes were amplified with the primer pairs listed in Table S2 in the supplemental material and were fused with a partial hygromycin resistance gene by fusion PCR to generate two DNA fragments (5′*MetB::HY/g* and *h/YG::3′MetB* for *AaMetB*, 5′*MetC::HY/g* and *h/YG::*3′*MetC* for *AaMetC*, and 5′*MetX::HY/g* and *h/YG::*3′*MetX* for *AaMetX*) overlapping the hygromycin phosphotransferase gene (HYG). To prepare protoplasts, fresh mycelia of A. alternata were inoculated in PDB liquid medium and cultured at 26°C on a rotary shaker for 48 h. The 2-day-old mycelia were chopped with a grinder, inoculated in 50 ml of PDB, and cultured on a rotary shaker at 26°C for another 36 h. These hyphae were filtered and collected with 3 layers of paper towels, rinsed several times with osmotic buffer, and cultured in a 150-ml Erlenmeyer flask containing 40 ml osmotic buffer amended with 1 g of snailase, 0.2 g of cellulase, and 0.2 g of lysozyme, respectively. The osmotic buffer was mixed with A. alternata, and resultant cell wall enzymes were incubated on a rotary shaker at 90 rpm at 26°C for 3 h and measured by hemocytometer under an optical microscope. Finally, the protoplasts of A. alternata were collected in STC buffer and stored at −80°C. For protoplast transformation, the two DNA fragments were transformed into wild-type protoplasts of A. alternata using CaCl_2_ and polyethylene glycol (PEG) as previously described (16). The transformants were recovered from the regeneration medium containing 100 μg/ml hygromycin. To identify transformants carrying the mutation, resistant transformants were screened by PCR using the inner and flanking primers of the hygromycin phosphotransferase gene and *AaMetB*, *AaMetC*, and *AaMetX* genes (YZ-MetB-1F and YZ-MetB-1R for *AaMetB*, YZ-MetC-1F and YZ-MetC-1R for *AaMetC*, and YZ-MetX-1F and YZ-MetX-1R for *AaMetX*). All the primers used in this study are listed in Table S2.

### Fungal growth assays and stress sensitivity tests.

The A. alternata wild-type strain Z7 and its derivative *ΔMetR*, *ΔMetB*, *ΔMetC*, and *ΔMetX* mutants were grown on PDA medium supplemented with 10 mM hydrogen peroxide (H_2_O_2_), 2 mM tert-butyl-hydroperoxide (t-BHP), 2 mM diethyl maleate (DEM), or 1 mM cumyl hydroperoxide (CHP); 3 mM l-methionine; 3 mM l-cysteine; fungicide (5 μg/ml chlorothalonil, 5 μg/ml propineb, 5 μg/ml thiophanate methyl, or 5 μg/ml difenoconazole); copper ion stress inducer (1 mM CuSO_4_); salt stress inducer (1 M NaCl); cell apoptosis inducer (5 μM camptothecin or 5 mM hydroxyurea); and fungal cell wall inhibitor (250 μg/ml Congo red). Each plate was inoculated with a 5-mm mycelial plug taken from the edge of a 5-day-old colony. The mycelial growth rate and colony morphology were measured after the plates were incubated at 25°C for 5 days. For the conidiation assay, mycelia of the 2-week-old cultures grown on PDA and V8 agar plates were resuspended in 5 ml sterile water and filtered with a layer of sterile cheesecloth. Conidial counts were performed using a hemocytometer mounted on a light microscope. Two independent strains of each gene mutant were used for all assays. All the experiments were performed at least twice, with three replicates of each treatment. To examine whether the mutant phenotype could be complemented by exogenous nutritional substances, *ΔMetR*, *ΔMetB*, *ΔMetC*, and *ΔMetX* mutants and the wild type were grown on PDA, V8, and MM supplemented with 10 mM l-methionine, l-cysteine, l-homocysteine, or l-glutathione.

### Virulence assays.

Fungal virulence was assessed on detached tangerine (Citrus reticulata Blanco) leaves inoculated by placing 5-mm mycelial plugs carrying fungal mycelium on tangerine leaves. To assess whether each fungal strain regained its virulence after supplementation with exogenous methionine, the fungal mutant was grown on PDA medium amended with 3.0 mM methionine for 3 to 4 days and then inoculated on tangerine leaves. Wild-type Z7 and the fungal mutants were grown on methionine-free PDA medium. To further investigate the loss of pathogenicity due to the inability of each mutant to penetrate the host cells, wound inoculation experiments were conducted on detached tangerine leaves. To further investigate whether the impaired virulence was due to methionine auxotrophy and/or cysteine auxotrophy, detached citrus leaves were inoculated with *ΔMetB*, *ΔMetC*, and *ΔMetX* supplemented with 3 mM l-methionine or l-cysteine. Each fungal strain was tested on at least 30 leaves, and the entire experiment was repeated twice. The inoculated leaves were placed in a humidified plastic box at 25°C for 2 to 4 days for lesion development.

### Transcriptome sequencing.

The mycelial plugs of 3-day-old colonies of A. alternata wild type or its derivative *AabZIP* mutants *ΔMetR*, *ΔMetB*, *ΔMetC*, and *ΔMetX* were grown in 150-ml Erlenmeyer flasks containing 50 ml liquid PDB medium and incubated in a rotary shaker at 25°C in the dark at 180 rpm for 2 days. To explore the ROS response, H_2_O_2_ was added to the wild type (WT) and *ΔMetR* to a final concentration of 10 mM with shaking for 2 h. The wounded citrus leaves were inoculated into liquid cultures of WT or *ΔMetR* for 2 h. For RNA extraction, the mycelium of each mutant strain was filtered, freeze-dried, frozen in liquid nitrogen, and ground into a fine powder using a mortar and pestle. Total RNA was extracted with the AxyPrep multi-source total RNA miniprep kit (Axygen, USA) according to the manufacturer’s instructions. The RNA purity was analyzed using the kaiaoK5500 spectrophotometer (Kaiao, Beijing, China). The Bioanalyzer 2100 platform (Agilent Technologies, CA, USA) was used to evaluate RNA integrity and concentration. RNA-Seq was performed on three biological replicates of each sample. RNA-Seq libraries were generated using an NEBNext Ultra RNA library prep kit for Illumina (catalog no. E7530L; NEB, USA). The RNA-Seq library concentration was measured using the Qubit RNA assay kit in Qubit 3.0. Insert size was assessed using the Agilent Bioanalyzer 2100 system, and qualified insert size was accurately quantified using the StepOnePlus real-time PCR system (valid concentration > 10 nM). Libraries were sequenced on the Illumina HiSeq platform, and 150-bp paired-end reads were generated.

### Comparative transcriptome analysis.

The whole genome of A. alternata strain Z7 was used as the reference genome (49). Trimmomatic v0.36 was used to remove adaptors and low-quality bases from the Illumina raw reads (50). The cleaned reads were subsequently mapped to the reference genome using HISAT2 v2.1.0 (51). To quantify gene expression levels, the software htseq-count was used to convert the mapped reads into a count matrix containing genes in each row and samples in each column (52). The R package DESeq2 was used to identify differentially expressed genes (DEGs) under the threshold of FDR ≤ 0.05 and absolute value of log_2_FC ≥ 1 (53). Gene ontology (GO) annotations and functional term mapping were performed using Blast2GO software (54). The DEGs were classified into GO categories using Web Gene Ontology Annotation Plot 2.0 (WEGO) (55). The secondary metabolite (SM) biosynthesis gene clusters were identified in the A. alternata Z7 genome by antiSMASH 5.0 (56). The DEGs and secondary metabolite gene clusters were visualized using Circos (57). Kyoto Encyclopedia of Genes and Genomes (KEGG) pathway analysis was performed for mapping enzymes to known metabolic pathways (58). The GO enrichment and KEGG pathway enrichment were performed using the R package clusterProfiler (59). The KEGG pathway was visualized using the Pathview R package (60). Weighted gene coexpression network analysis (WGCNA) was performed using the R package WGCNA to explore the pattern of genetic associations and synergistically altered gene sets among transcriptomes of *ΔMetR*, *ΔMetB*, *ΔMetC*, *ΔMetX*, and wild-type strain Z7 (61). The coexpression gene network was visualized using the ggnet R package.

### Quantitative RT-PCR.

To verify the transcriptome data obtained by RNA-Seq, quantitative reverse transcription-PCR (qRT-PCR) was performed in the Bio-Rad CFX96 real-time PCR detection system (Bio-Rad, USA). The fungus A. alternata wild-type Z7 and *ΔMetR* mutants were cultured in 150-ml Erlenmeyer flasks containing 50 ml of liquid PDB medium and incubated on a rotary shaker at 25°C and 180 rpm in the dark for 2 days. For RNA extraction, the mycelia of the *ΔMetR* mutant and wild type were immediately filtered through four layers of cheesecloth, freeze-dried, and ground into a fine powder in liquid nitrogen. Fungal RNA was extracted from each sample using the AxyPrep multisource total RNA miniprep kit (Axygen Biotechnology, Hangzhou, China) according to the manufacturer’s instructions. In total, 5 μg RNA was reverse transcribed into cDNA using the HiScript II Q RT SuperMix kit (Vazyme Biotech Co., Ltd., Nanjing, China). Real-time quantitative PCR was performed using the ChamQ SYBR qPCR master mix kit (Vazyme Biotech Co., Ltd., Nanjing, China). The Bio-Rad CFX96 real-time PCR detection system was used for qRT-PCR with an initial denaturation at 95°C for 30 s, followed by 40 cycles of denaturation at 95°C for 10 s and then annealing and extension at 60°C for 30 s. For each sample, all treatments were performed in triplicate. In this study, the A. alternata actin gene was used as an endogenous control.

### Data availability.

All the RNA-Seq Illumina data have been deposited at NCBI under the BioProject accession no. PRJNA655610. All gene sequences and proteomes are available in the Zenodo repository at https://doi.org/10.5281/zenodo.4173635. The figures, R codes, and custom Perl scripts are available on the figshare repository at https://doi.org/10.6084/m9.figshare.13174619.

## Supplementary Material

Supplemental file 1

Supplemental file 2
