# Supplementary material for: Critical Role of MetR/MetB/MetC/MetX in Cysteine and Methionine Metabolism, Fungal Development, and Virulence of Alternaria alternata
Source: Appl Environ Microbiol. 2021 Jan 29;87(4):e01911-20. doi: 10.1128/AEM.01911-20 (PMC7851696; doi:10.1128/AEM.01911-20)
Supplement: Supplemental file 2 [file AEM.01911-20-s0002.pdf]

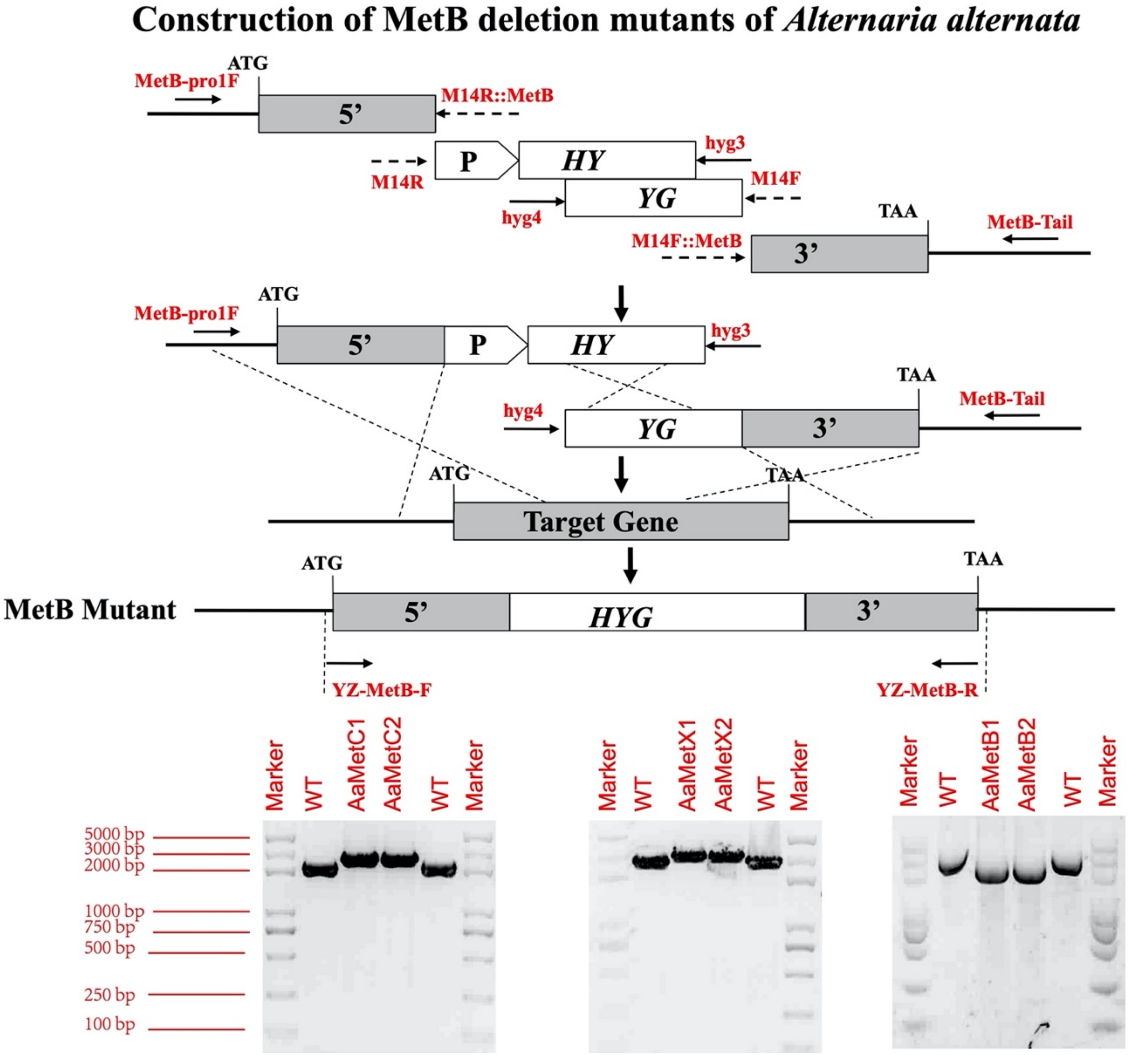

FIG. S1 Verification of the positive bZIP transformants by polymerase chain reaction (PCR). (A) *ΔMetC* mutant (MetC1, MetC2); (B) *ΔMetX* mutant (MetX1, MetX2); (C) *ΔMetB* mutant (MetB1, MetB2).

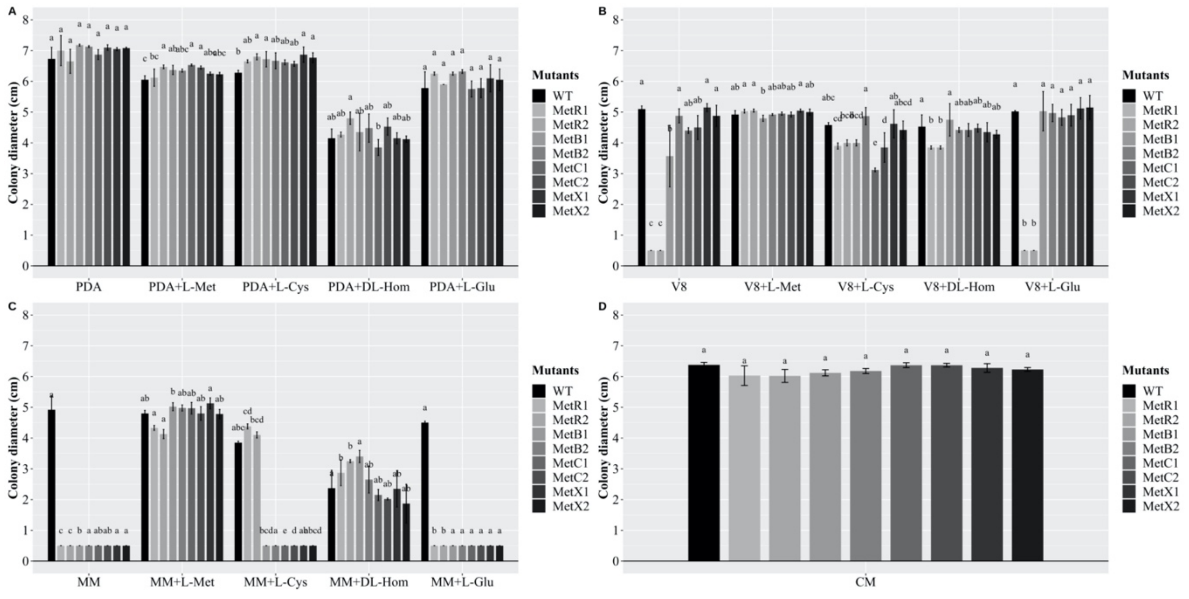

FIG. S2 Bar plot showing *AaMetB*, *AaMetC*, and *AaMetX* are required for methionine metabolism, while *AaMetR* are required for cysteine metabolism. Growth of the wildtype strain, *ΔMetR*, *ΔMetB*, *ΔMetC* and *ΔMetX* on PDA, V8, and MM medium supplemented with 3 mM L-methionine, 3 mM L-cysteine, 3 mM L-homocysteine, or 3 mM glutathione.

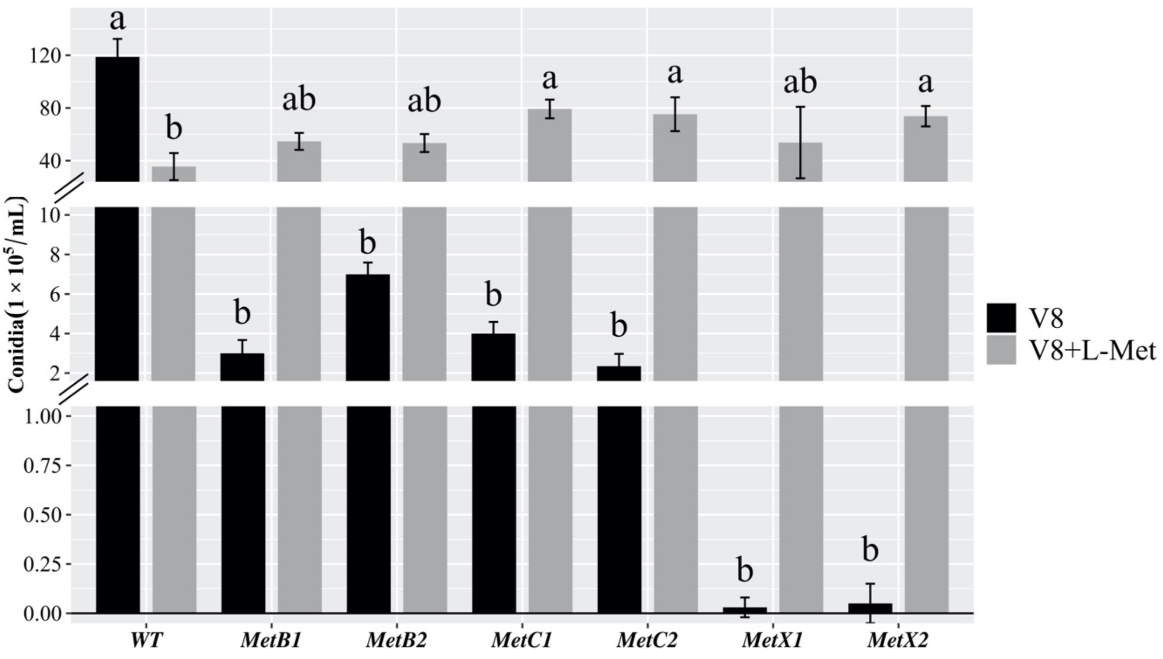

FIG. S3 Bar plot showing *AaMetB*, *AaMetC*, and *AaMetX* is required for conidiation of *A. alternata*. The conidia production of wildtype strain (Z7), *ΔMetB* mutant (MetB1, MetB2), *ΔMetC* mutant (MetC1, MetC2), *ΔMetX* mutant (MetX1, MetX2) on V8 medium, and V8 medium supplemented with L-Methionine were calculated by a hemocytometer under an optical microscope.

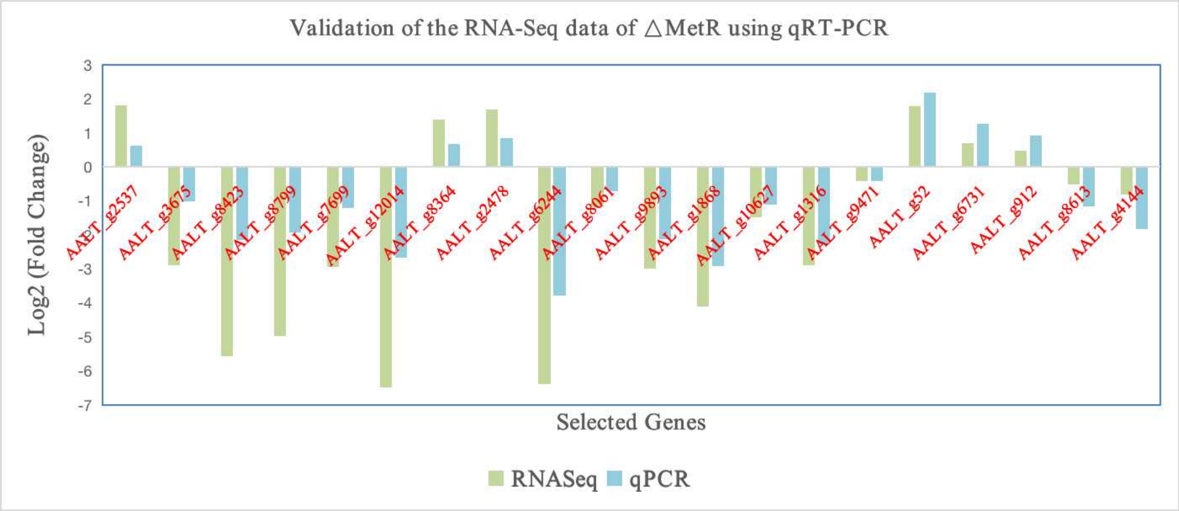

FIG. S4 Validation of the RNA-seq data of *ΔMetR* mutant using qRT-PCR.

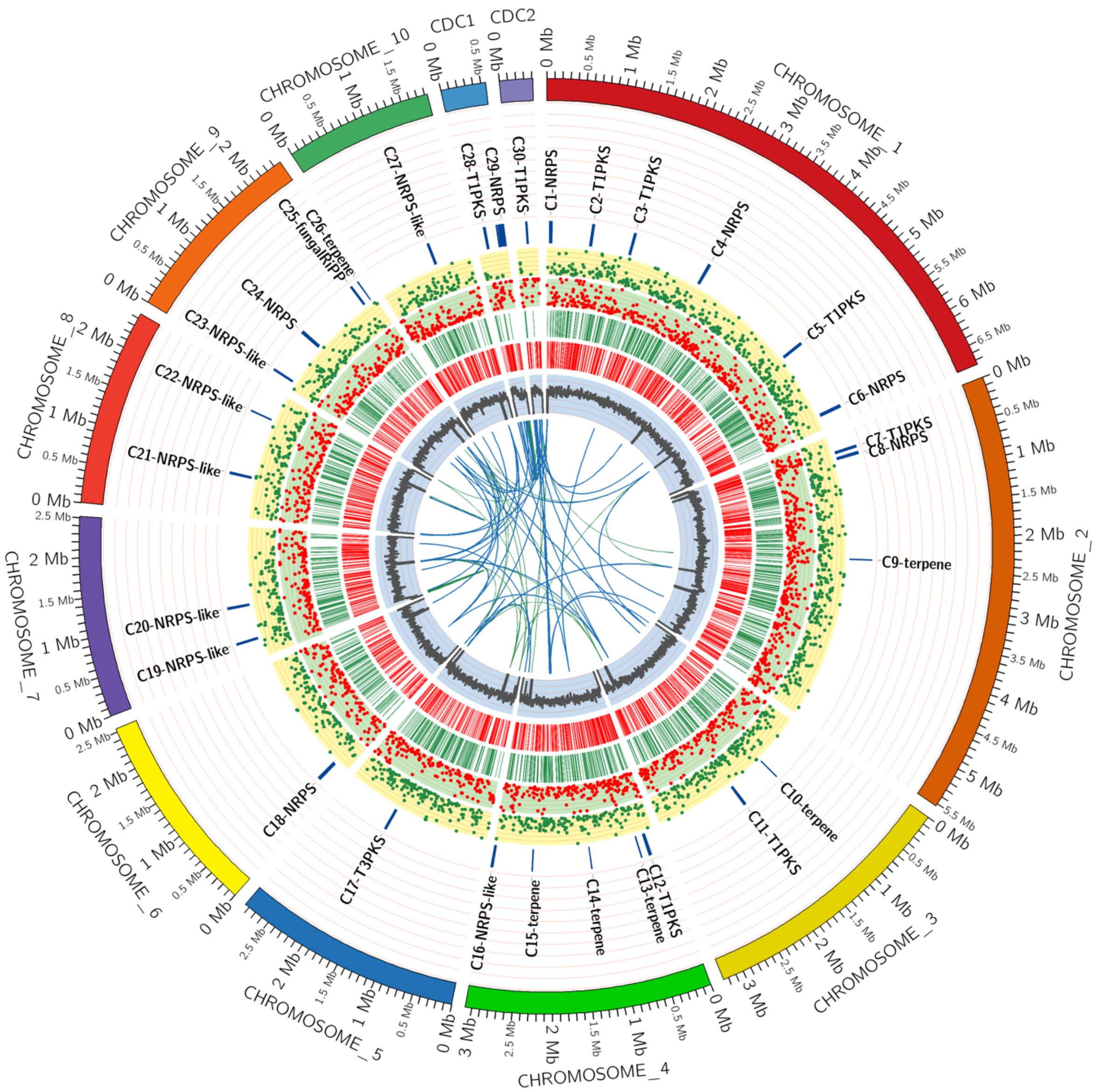

FIG. S5 Circos plot displaying the differences in gene expression in *ΔMetB* compared with wild-type strain Z7. Each circle from the periphery to the core represents the following: chromosomal location; secondary metabolite gene clusters; differentially expressed genes (DEGs); GC content; Gene duplications are shown in the center.

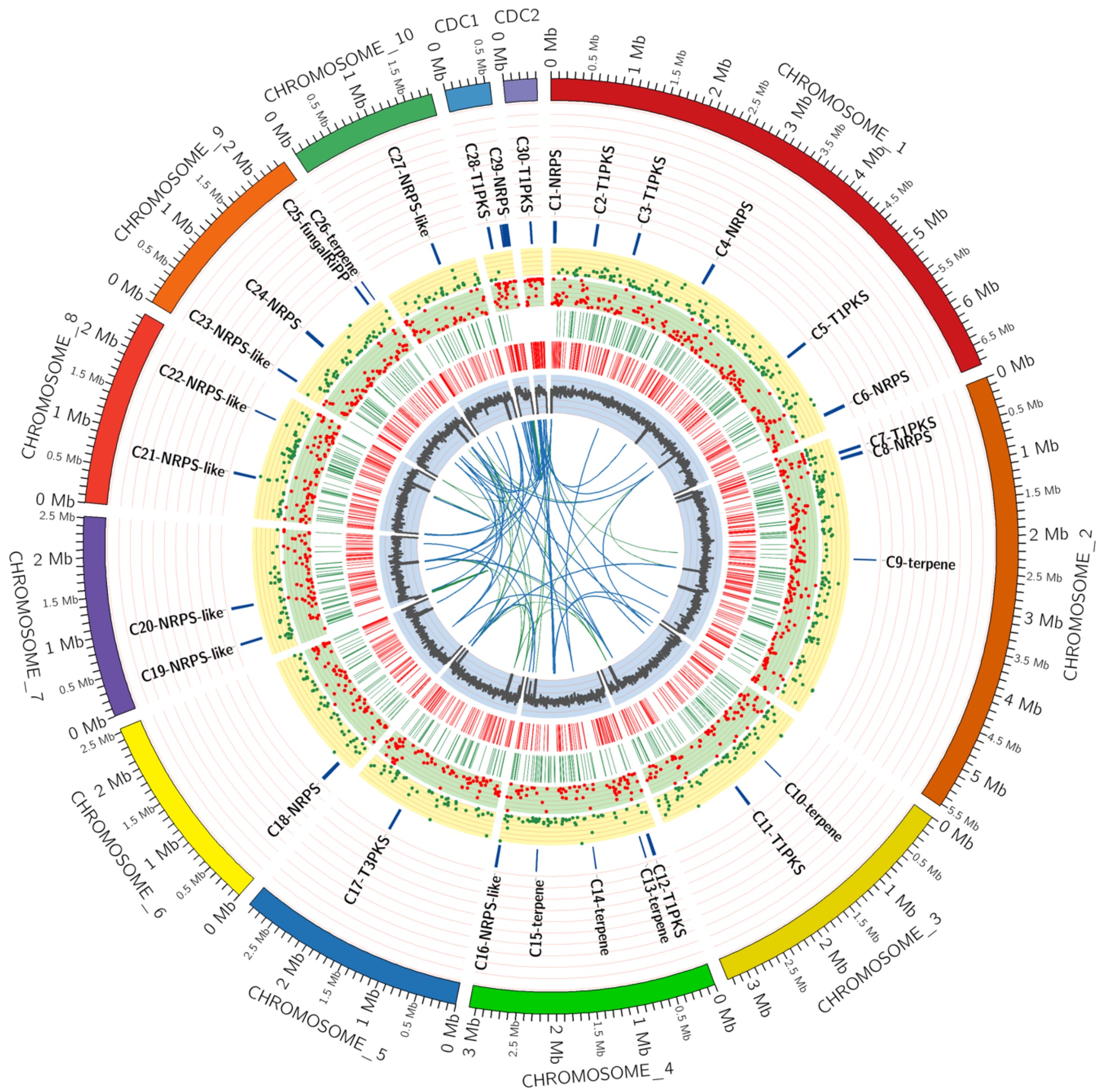

FIG. S6 Circos plot displaying the differences in gene expression in *ΔMetC* compared with wild-type strain Z7.

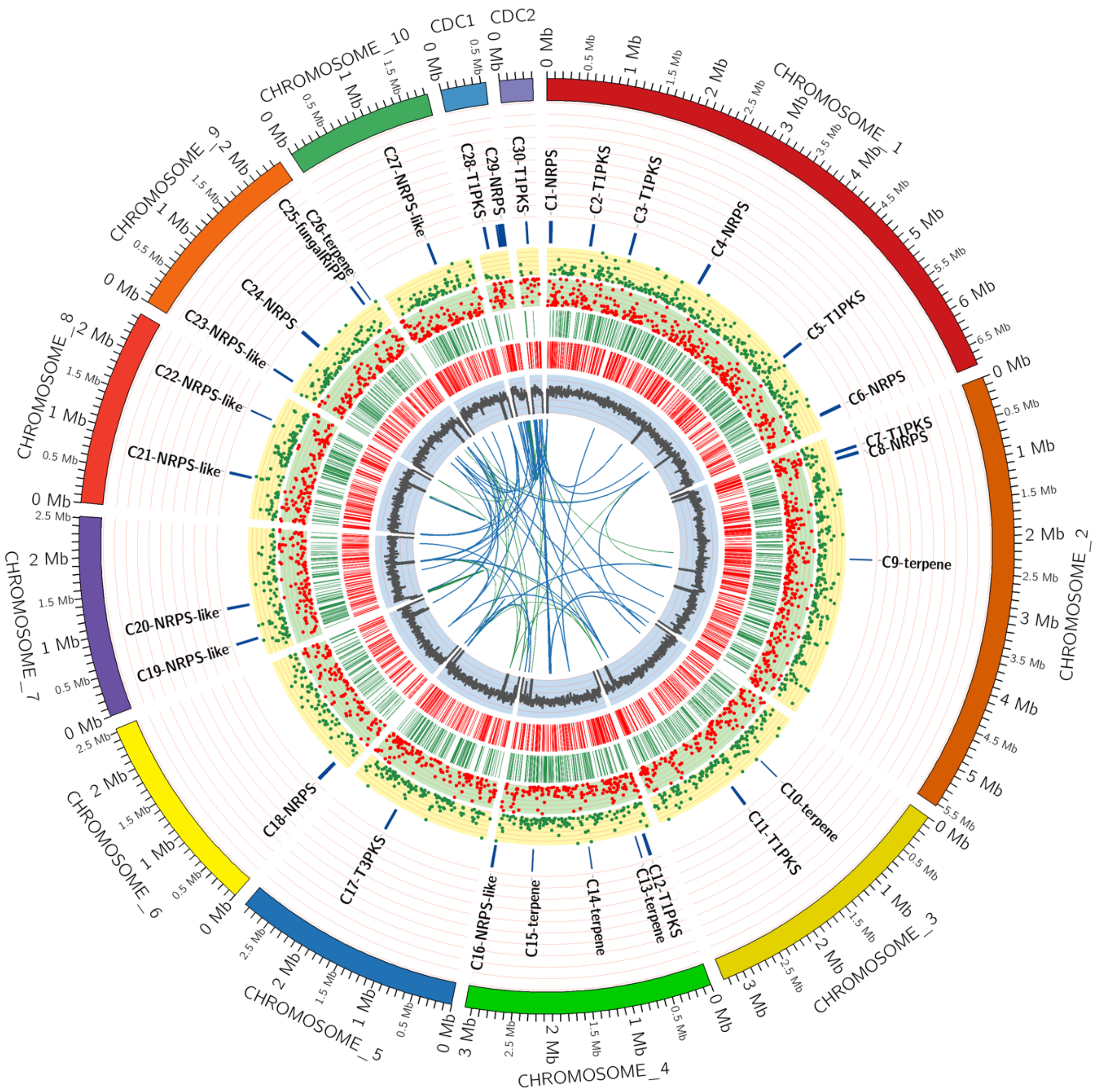

FIG. S7 Circos plot displaying the differences in gene expression in *ΔMetX* compared with wild-type strain Z7.

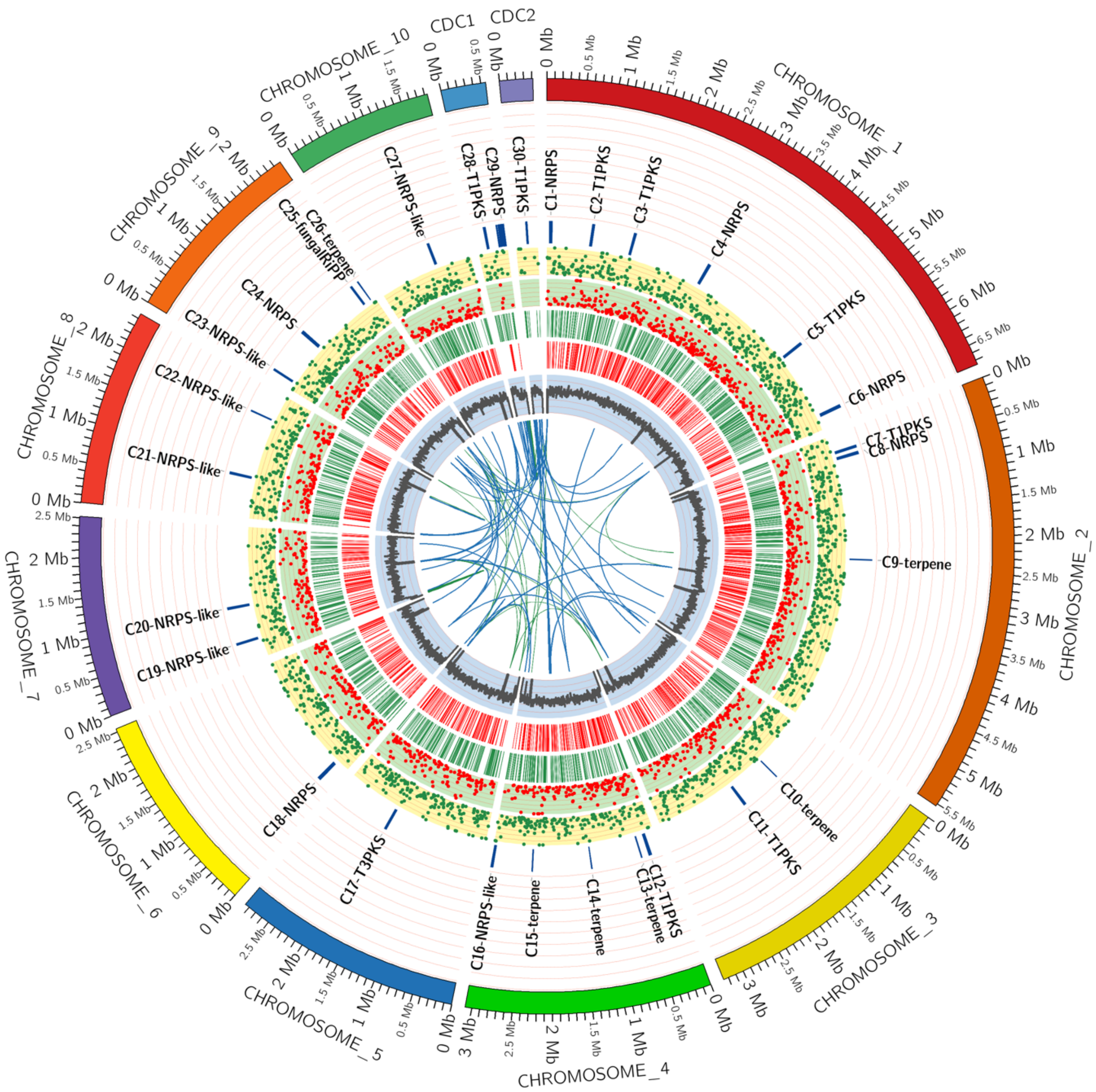

FIG. S8 Circos plot displaying the differences in gene expression in *ΔMetR* compared with wild-type strain Z7.

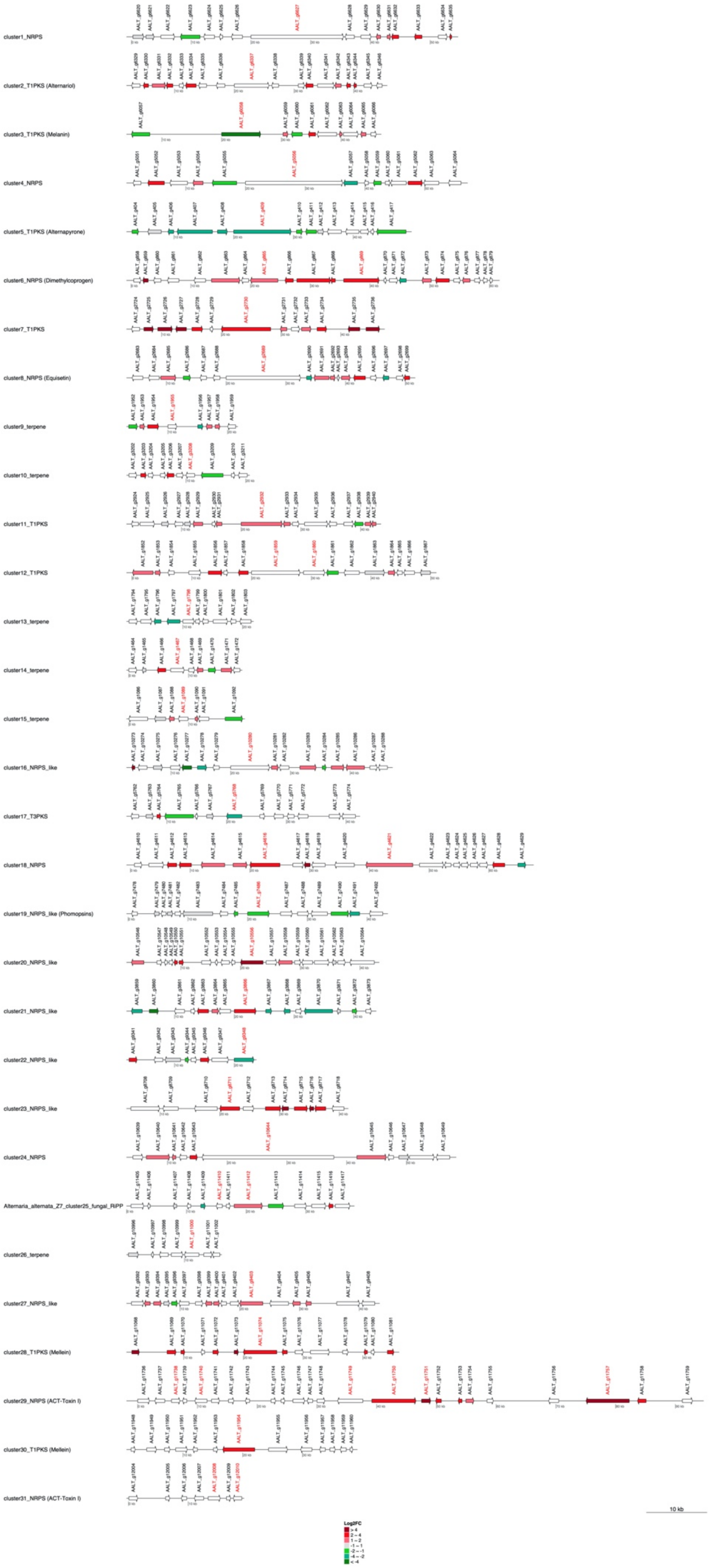
cluster1_NRPS
cluster2_T1PKS (Alternariol)
cluster3_T1PKS (Melanin)
cluster4_NRPS
cluster5_T1PKS (Alternapyrone)
cluster6_NRPS (Dimethylcoprogen)
cluster7_T1PKS
cluster8_NRPS (Equisetin)
cluster9_terpene
cluster10_terpene
cluster11_T1PKS
cluster12_T1PKS
cluster13_terpene
cluster14_terpene
cluster15_terpene
cluster16_NRPS_like
cluster17_T3PKS
cluster18_NRPS
cluster19_NRPS_like (Phomopsins)
cluster20_NRPS_like
cluster21_NRPS_like
cluster22_NRPS_like
cluster23_NRPS_like
cluster24_NRPS
Alternaria_alternata_Z7_cluster25_fungal_RiPP
cluster26_terpene
cluster27_NRPS_like
cluster28_T1PKS (Mellein)
cluster29_NRPS (ACT-Toxin I)
cluster30_T1PKS (Mellein)
cluster31_NRPS (ACT-Toxin I)
10 kb
Log2FC
> 4
2 ~ 4
1 ~ 2
-1 ~ 1
-2 ~ -1
-4 ~ -2
< -4

FIG. S9 The gene expression patterns of SM Gene Clusters in the transcriptome of *ΔMetB* mutant, the color of each gene indicates the Log2FC of each gene.

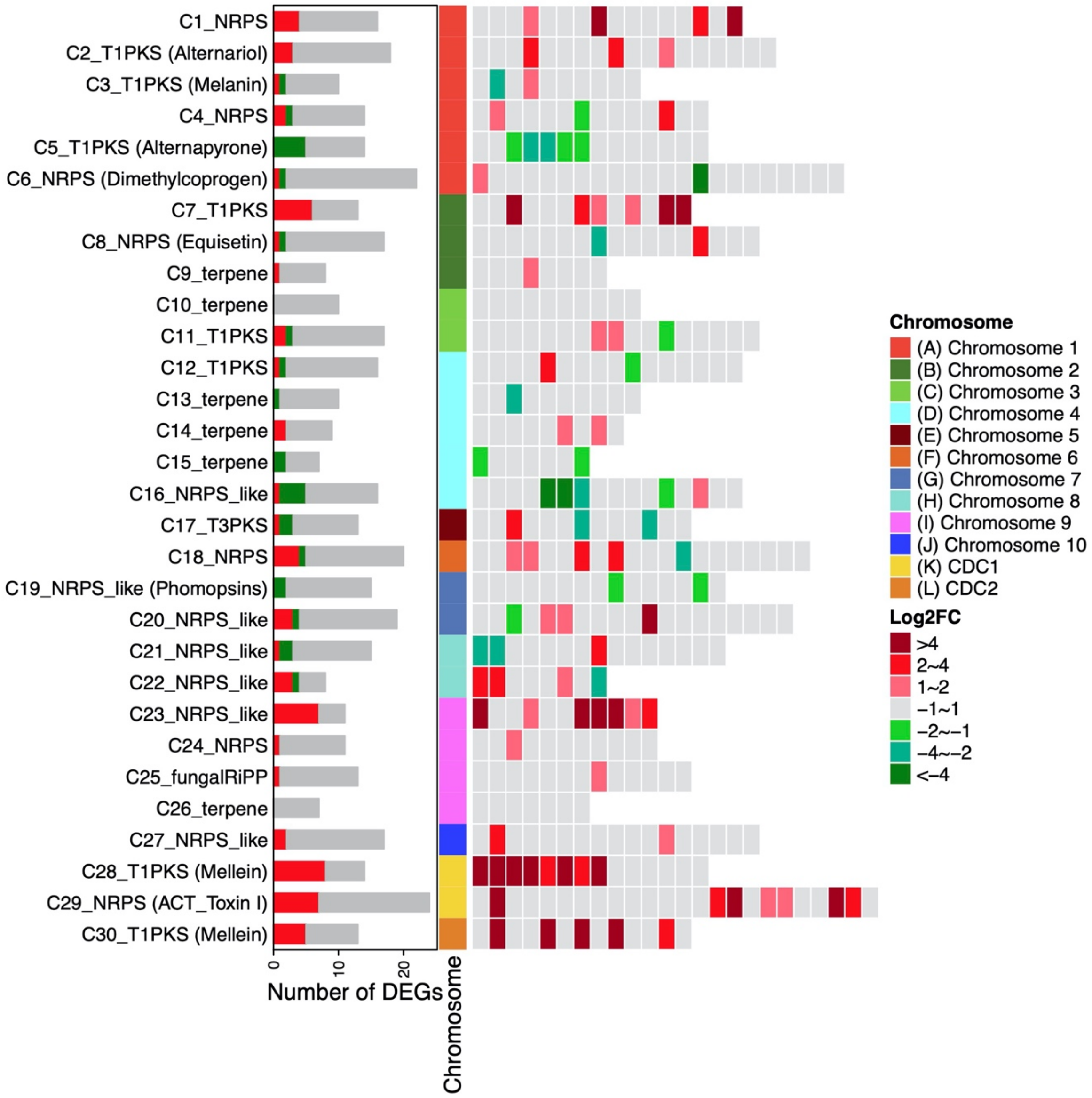

FIG. S10 The gene expression patterns of SM Gene Clusters in the transcriptome of *ΔMetC* mutant, the color of each gene indicates the Log2FC of each gene.

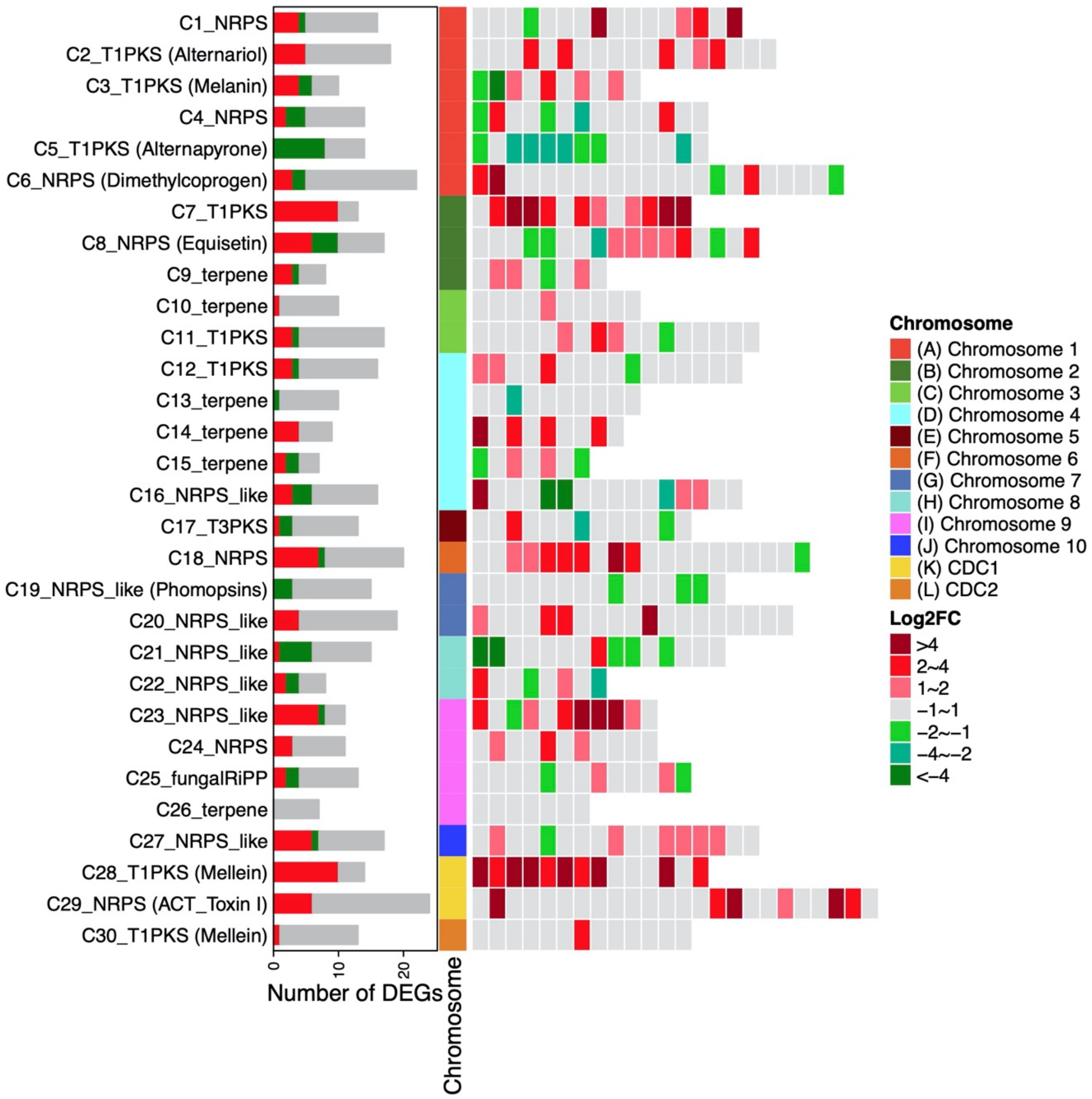

FIG. S11 The gene expression patterns of SM Gene Clusters in the transcriptome of *ΔMetX* mutant, the color of each gene indicates the Log2FC of each gene.

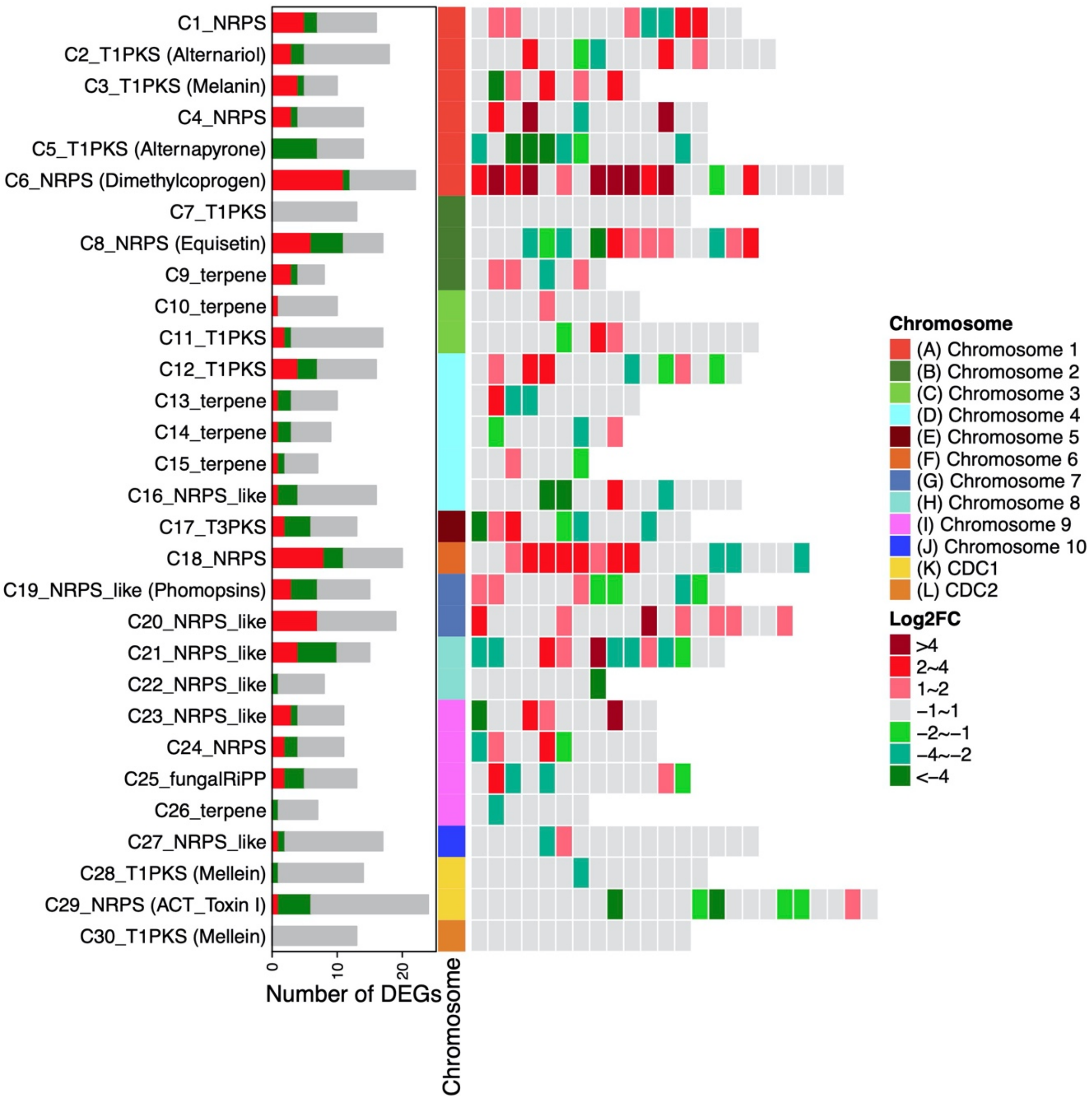

FIG. S12 The gene expression patterns of SM Gene Clusters in the transcriptome of *ΔMetR* mutant, the color of each gene indicates the Log2FC of each gene.

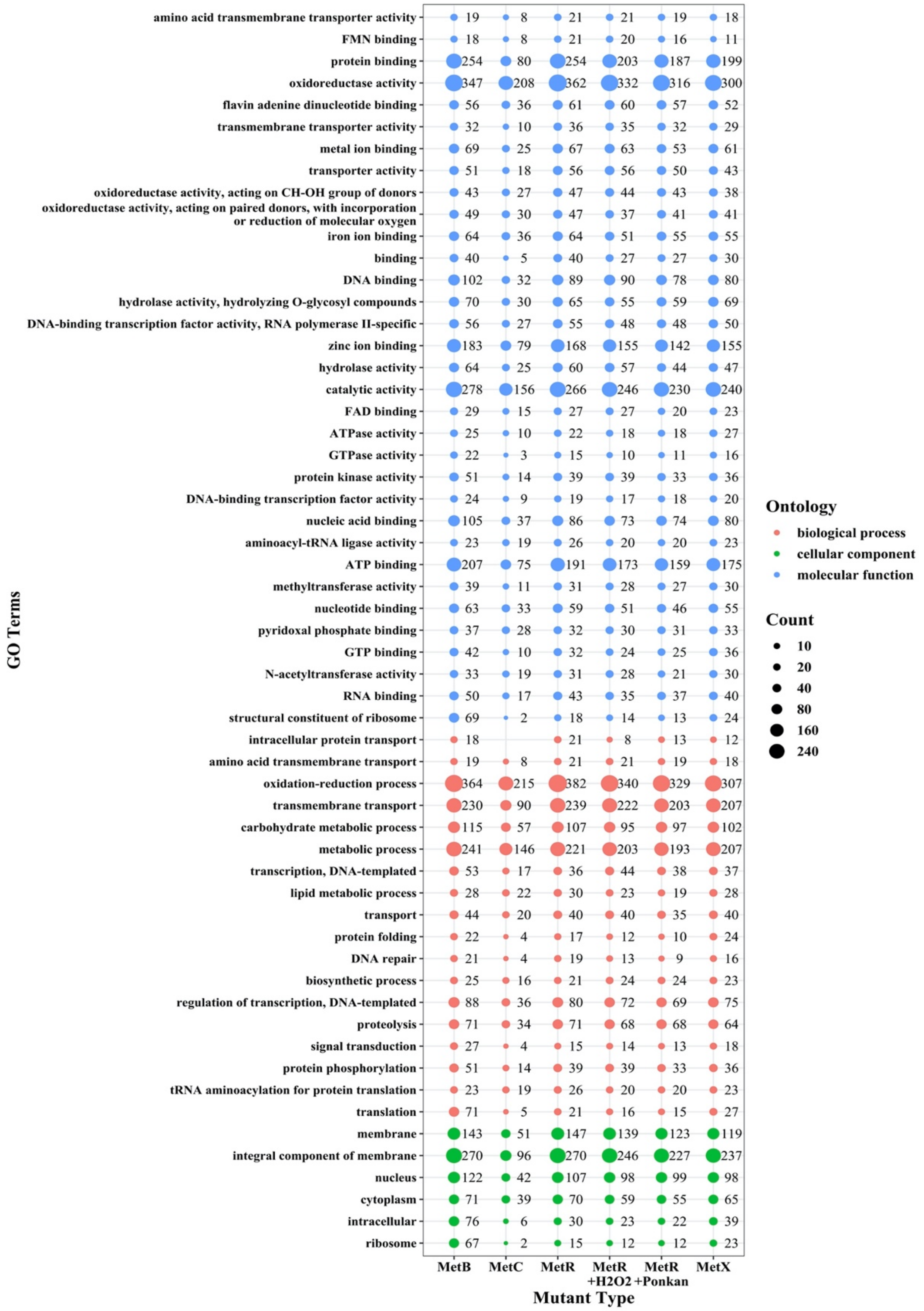

FIG. S13 Comparative Gene Ontology (GO) analysis of the differential expressed genes (DEGs) between the transcriptome of *ΔMetB*, *ΔMetC*, *ΔMetX*, *ΔMetR* mutant and wild-type strain *Z7*. The results are summarized in three main GO categories (cellular component, molecular function and biological process). The x-axis indicates different mutants. The y-axis indicates the GO term.

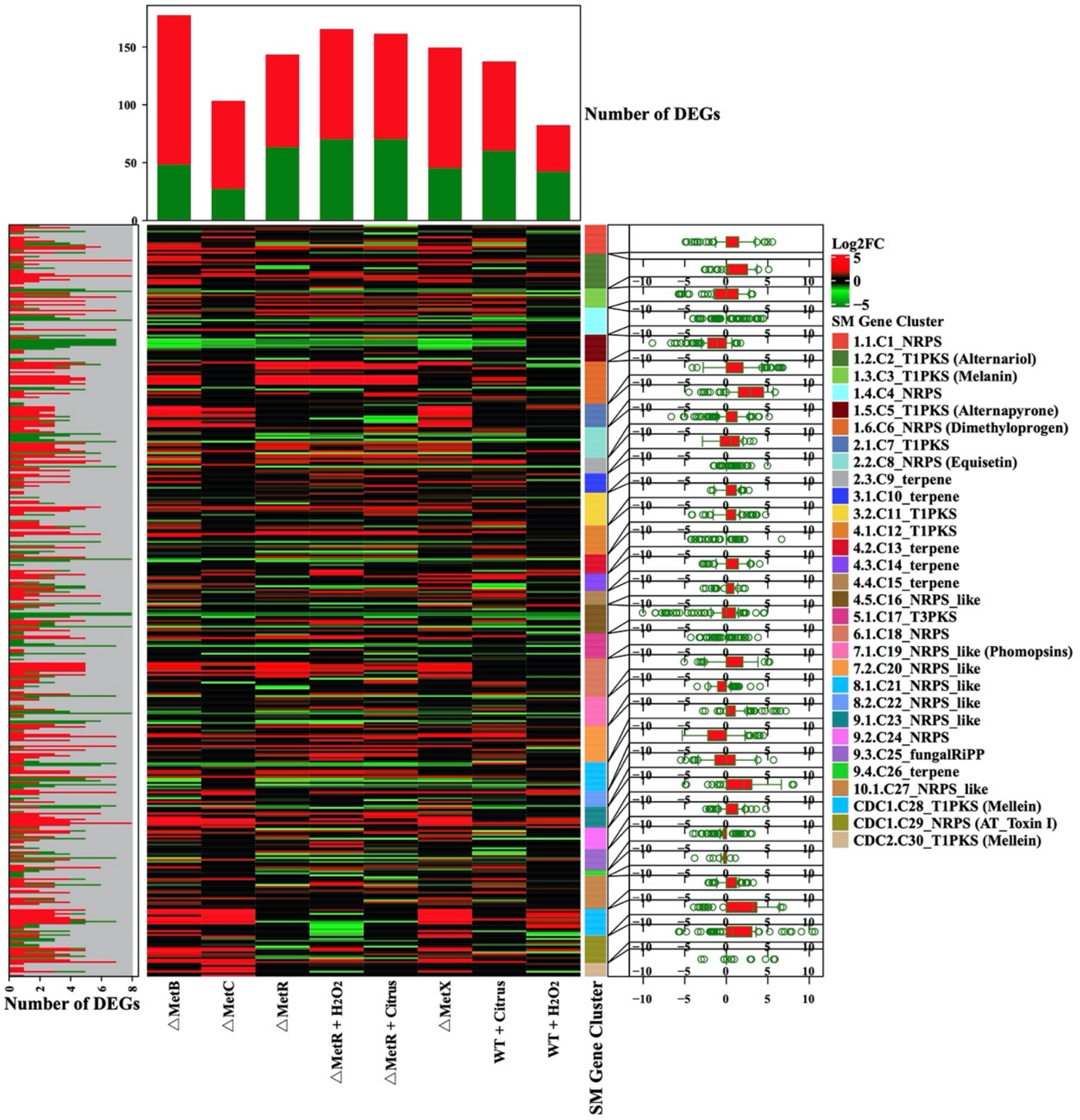

FIG. S14 Heatmap of differentially expressed genes (DEGs) enriched in SM Gene Clusters reveal differences in expression between wild-type and mutants *ΔMetB, ΔMetC, ΔMetX, ΔMetR* in PDB medium, *ΔMetR* supplemented with H_2O_2, *ΔMetR* inoculated with citrus leaves. Bar plot describing the differential expressed SM genes of each mutant and the wild-type under different conditions. The bar plot above the heat map shows the differentially expressed genes (DEGs) of each treatment. The bar plot to the right indicates the differentially expressed genes (DEGs) of each gene. The color of red indicates upregulation; black, no differential expression; green, downregulation. The color bar annotation on the left shows the indicated SM Gene Clusters.

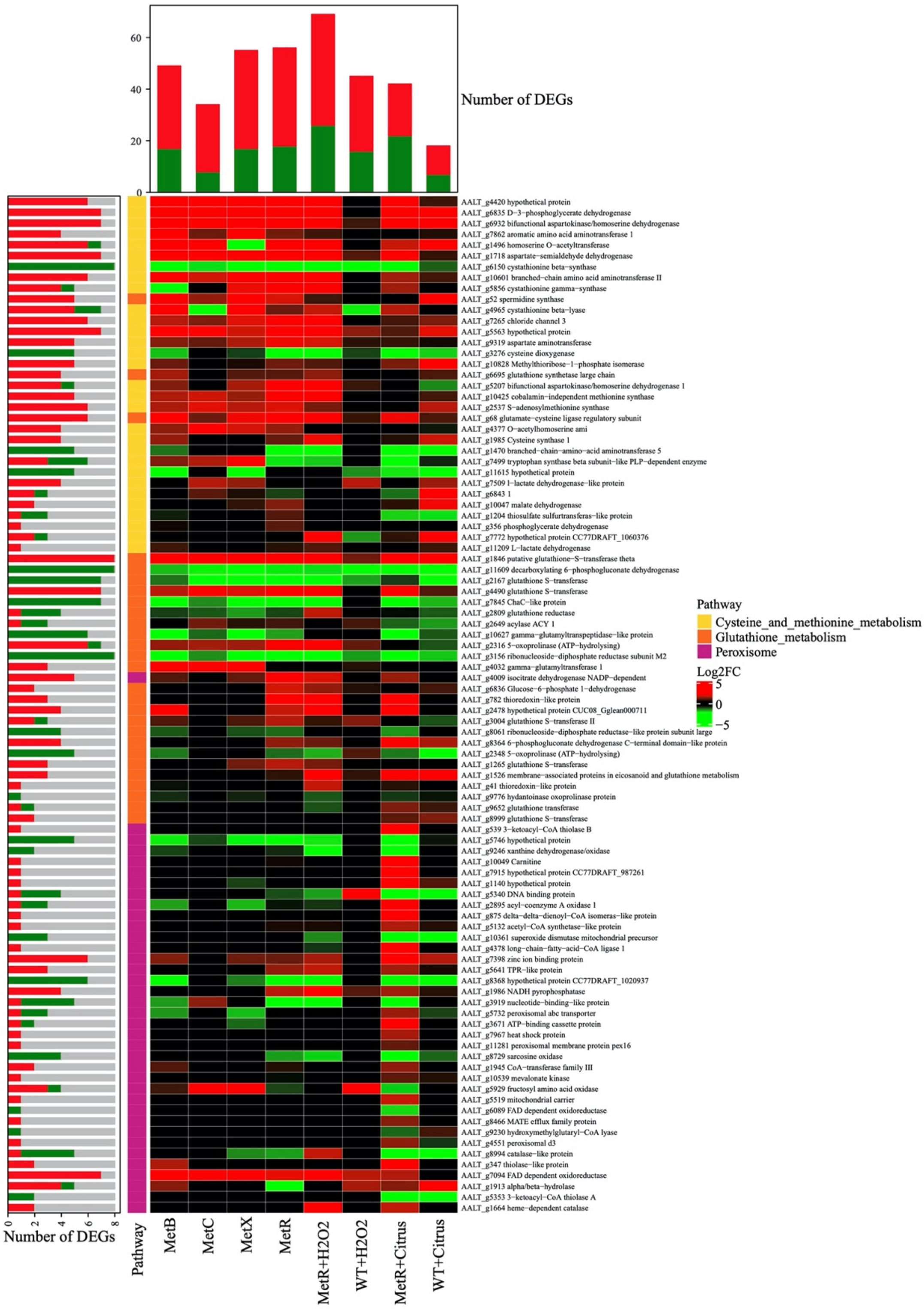

FIG. S15 Heatmap of differentially expressed genes (DEGs) enriched in the "cysteine and methionine metabolism", "glutathione metabolism", "proteasome" reveal differences in expression between wild-type and mutants *ΔMetB, ΔMetC, ΔMetX, ΔMetR* in PDB medium, *ΔMetR* supplemented with H_2O_2, *ΔMetR* inoculated with citrus leaves. The histogram above the heat map shows the differentially expressed genes (DEGs) of each treatment. The histogram

on the left of the heatmap indicates the number of differentially expressed genes (DEGs) of each SM gene. The gene name on the right of the heatmap shows the gene name and the functional annotation from NR database. The expression level of each gene relative to the mean across all the experimental conditions on the log scale has been represented as follows: red indicates upregulation; gray, no differential expression; green, downregulation.

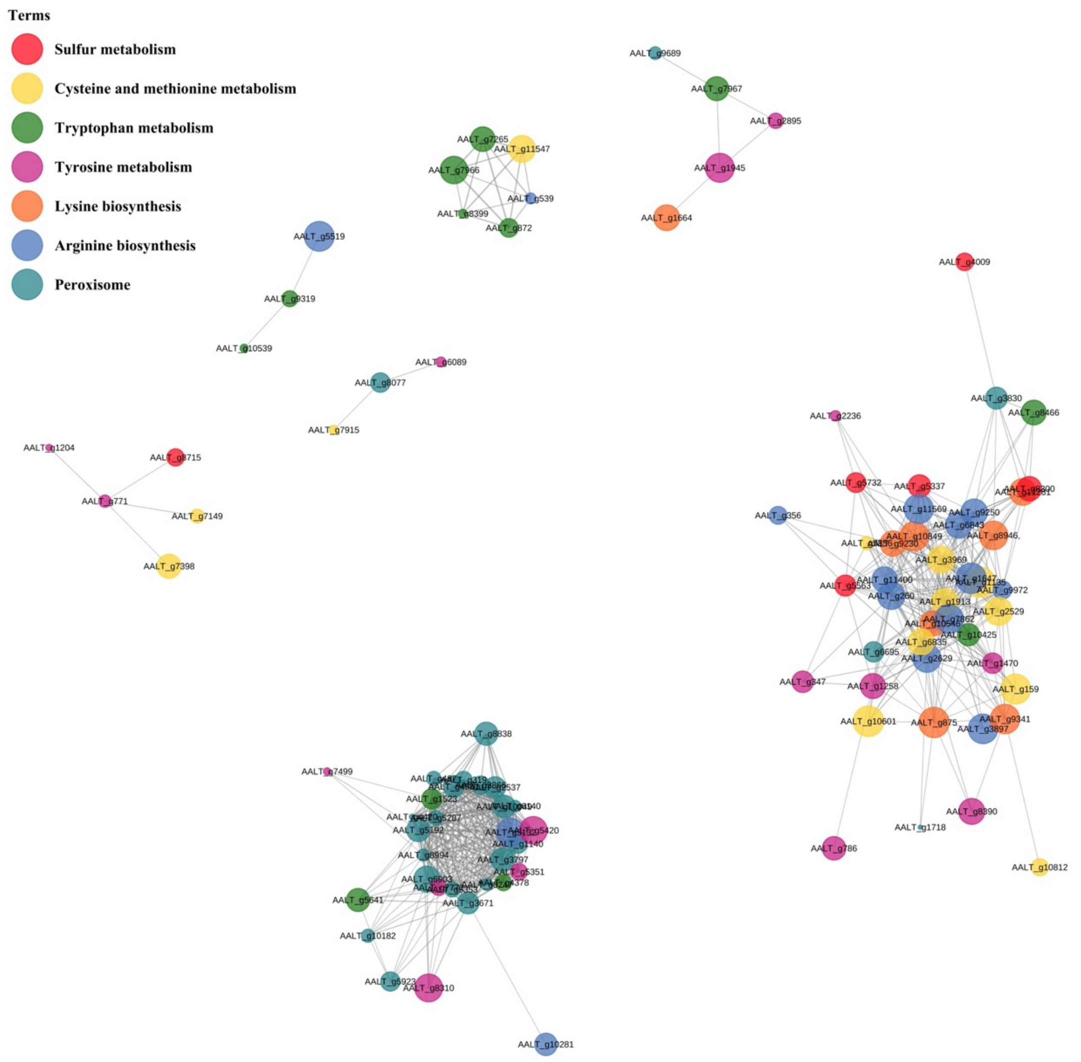

FIG. S16 Weighted gene co-expression network analysis (WGCNA) of the genes related to "sulfur metabolism", "cysteine and methionine metabolism", "tryptophan metabolism", "tyrosine metabolism", "lysine biosynthesis", "arginine biosynthesis", "peroxisome" in transcriptome profiles. The threshold of the weight between each gene were >=0.3 and the color of each gene indicates different KEGG Pathway terms. The gene size in the network indicates the relative expression of each gene.
